# Exploring the Chemical Space of *Paenibacillus* NRPs and Discovery of Paenilipoheptin B

**DOI:** 10.1021/acs.orglett.5c00231

**Published:** 2025-03-14

**Authors:** Nataliia V. Machushynets, Vladyslav Lysenko, Chao Du, Cornelis J. Slingerland, Somayah S. Elsayed, Mark R. Liles, Nathaniel I. Martin, Gilles P. van Wezel

**Affiliations:** † Molecular Biotechnology Group, Institute of Biology, Leiden University, Sylviusweg 72, 2333 BE Leiden, The Netherlands; ‡ Biological Chemistry Group, Institute of Biology, Leiden University, Sylviusweg 72, 2333 BE Leiden, The Netherlands; § Department of Biological Sciences, Auburn University, 120 W Samford Ave, Auburn, Alabama 36849, United States; ∥ Department of Microbial Ecology, Netherlands Institute of Ecology, Droevendaalsesteeg 10, 6708 PB Wageningen, The Netherlands

## Abstract

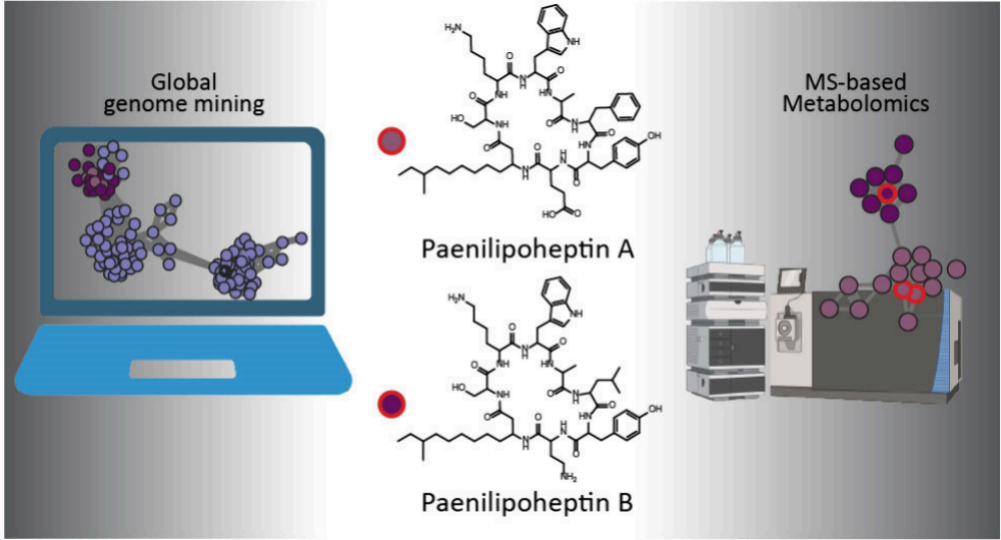

A combination of genomic and metabolomic analyses paired with molecular networking was applied to a collection of *Paenibacillus* spp. to identify the producers of a little-studied class of lipopeptides known as paenilipoheptins. Mass spectrometry and NMR spectroscopy allowed revision of the structure of previously reported paenilipoheptin A and elucidation of the structure of novel paenilipoheptin B.

The rise in antimicrobial resistance (AMR) has led to an increased interest in novel antibiotics. An important class of natural products with therapeutic potential produced by bacteria is nonribosomal peptides (NRPs).^1^ NRPs currently used in the clinic, such as bacitracin, daptomycin, polymyxin, and vancomycin, constitute effective treatments for infections caused by multidrug-resistant pathogens, though also for these compounds AMR becomes an issue.^2,3^ A wide variety of bacteria produce NRPs,^4^ whereby *Paenibacillus* spp. have yielded several potent antimicrobial lipopeptides, such as polymyxins, tridecaptins, paenibacterins, octapeptins, and pelgipeptins, among others.^5,6^ We previously bioinformatically analyzed 785 complete genomes from *Paenibacillus* spp. to identify biosynthetic gene clusters (BGCs) that encode the biosynthesis of nonribosomal peptide synthetases (NRPSs).^7^ NRPSs are large multifunctional enzymes that have modular structures, with each NRPS module catalyzing the incorporation of a specific substrate into the growing peptide.^8,9^ A typical module consists of three enzymatic domains, namely, adenylation (A), thiolation (T), condensation (C), and epimerization (E) domains and the terminal thioesterase (TE).^10^ The collinearity rule of NRPS systems, combined with knowledge of the specificity-conferring code of the A domain, allows for prediction of the peptide structures synthesized by the corresponding NRPS.

To visualize the diversity, distribution, and NRPS novelty, a sequence similarity network (SSN) was constructed using BiG-SCAPE.^11^ Besides known classes of BGCs for among others polymyxins, tridecaptins, fusaricidins, paenibacterins, octapeptins, bacitracins, and cilagicins,^7^ the analysis also identified BGCs for unknown or partially characterized classes of NRPs. The paenilipoheptins are notable examples (Figure 1A). While paenilipoheptin A was detected in the extracts of *P. polymyxa* E681 using LC-MS, the compound itself had not been isolated, and thus, the specific bioactivity was also unknown.^12^

**Figure 1:**
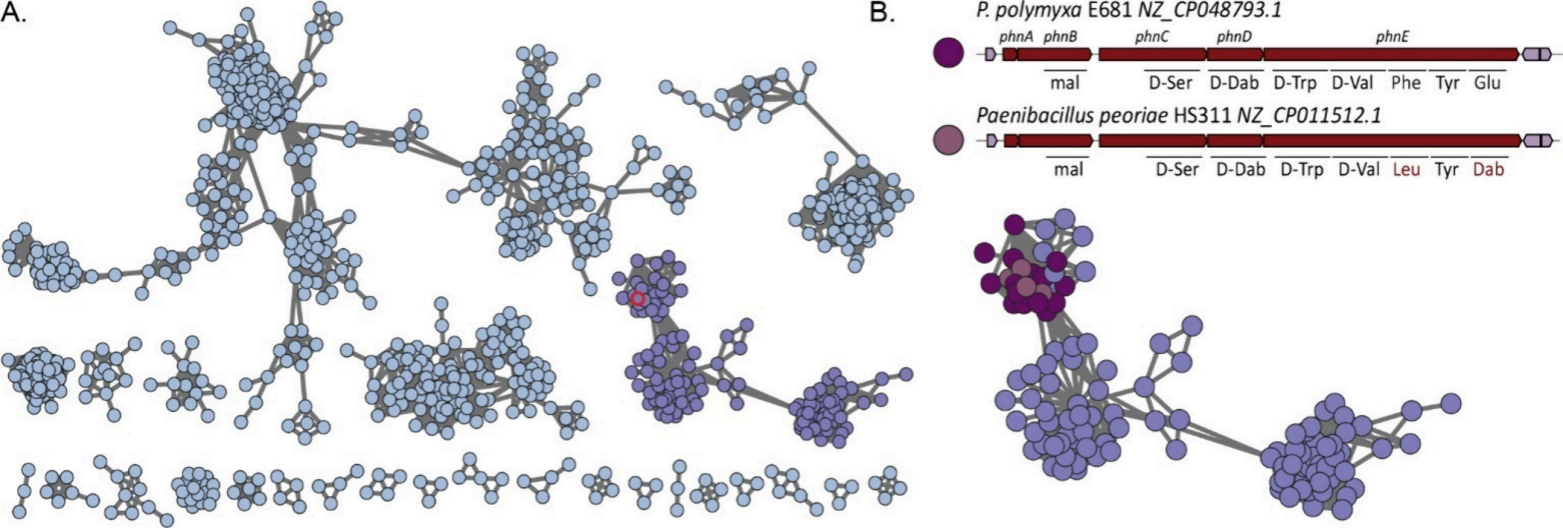
Sequence similarity network of *Paenibacillus* NRPS BGCs and predicted paenilipoheptin BGCs. (A) BiG-SCAPE sequence similarity network (SSN) (c 0.25) containing validated NRPS BGCs of *Paenibacillus* spp. visualized in Cytoscape. Each node represents one NRPS BGC predicted by antiSMASH. Singletons and single links are not shown. The node corresponding to the paenilipoheptin BGC from *P. polymyxa* E681 is emphasized in red. (B) Expanded view of gene cluster families (GCFs) predicted to specify paenilipoheptin-like compounds. The BGCs and predicted amino acid sequences for paenilipoheptin A and the newly identified paenilipoheptin B are shown above the network. Notable differences in the NRPS assembly lines of paenilipoheptin B compared to paenilipoheptin A are highlighted in modules 6 and 8, shown in red.

The paenilipoheptin BGC of *P polymyxa* E681 encodes a hybrid NRPS-Trans-AT-PKS that produces paenilipoheptin A.^12^ The entire paenilipoheptin assembly line involves three peptide synthetases, PhnC, PhnD, and PhnE (Figure 1B). The one-module enzymes PhnC and PhnD were predicted to mediate the incorporation of amino acids Ser1 and Dab2, respectively. PhnE consists of five modules with predicted substrate specificity of A domains for the amino acids Trp3, Val4, Phe5, Tyr6, and Glu7. Modules 2–5 contain epimerization domains, indicating that Ser1, Dab2, Trp3, and Val4 may be converted to the d-configuration. The SSN highlighted numerous BGCs with architectures similar to the paenilipoheptin BGCs found in the genome of *P. polymyxa* E681. To predict the amino acid sequences of paenilipoheptins encoded by these BGCs, an in silico analysis of A-domain substrate specificities was conducted using antiSMASH 7.1.0.^13^ Based on the predicted amino acid sequences, we classified the detected paenilipoheptin-like BGCs into two groups.

The first group consisted of paenilipoheptin BGCs with predicted amino acid sequences identical with those of paenilipoheptin A from *P. polymyxa* E681, with the predicted sequence d-Ser–d-Dab–d-Trp–d-Val–l-Phe–l-Tyr–l-Glu (Figure 1B). The second group included BGCs with variations at amino acid positions 5 and 7, resulting in the predicted sequence d-Ser–d-Dab–d-Trp–d-Val–l-Leu–l-Tyr–l-Dab (Figure 1B). This variation suggests the production of a new analogue, designated paenilipoheptin B, thereby expanding the diversity of known paenilipoheptins. Sequence similarity network indicates that paenilipoheptins were mainly produced by species of *P. polymyxa*, *P. peoriae*, and *P. jamilae.* Therefore, a subset of strains from the Auburn University (USA) Plant-Associated Microbial strain collection, showing high 16S rRNA gene sequence similarity (greater than 99%) with these species, was selected to identify potential paenilipoheptin producers. Isolates were cultured in TSA media in 96 deep-well plates for 72 h and extracted with isopropyl alcohol (IPA) supplemented with 0.1% (v/v) formic acid (FA). The extracts were then subjected to LC-MS/MS analysis to detect the produced secondary metabolites. LC-MS/MS data were processed with MzMine 2^14^ and exported for GNPS FBMN.^15^ FBMN analysis resulted in a molecular network consisting of 514 parent ions (nodes) connected through 738 edges (Figure 2A). Characteristics of the natural products, such as annotation, *m*/*z* value, and species-specific molecule production were visualized using Cytoscape 3.9.1.^16^ Mass spectrometry-based molecular networking allows clustering of molecules with similar MS/MS fragmentation patterns, which stem from the similarity in their structures.^17^ We focused on a molecular family of compounds with a node of *m*/*z* 562.3133, which corresponds to the exact mass of the previously reported paenilipoheptin A (Figure 2A).^12^

**Figure 2:**
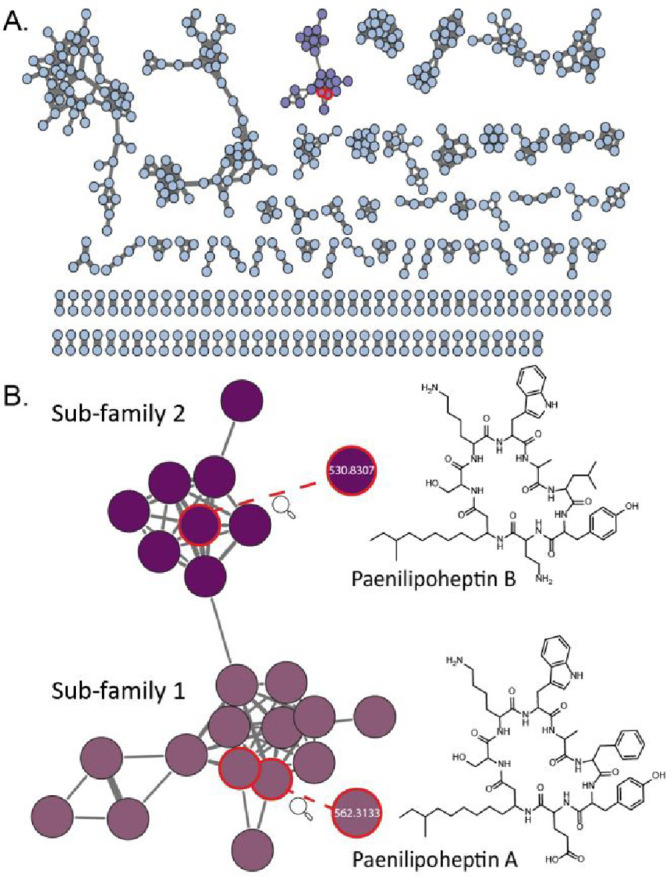
Molecular network of the mass features detected in the bacterial extracts from a subset of 25 plant-associated *Paenibacillus* isolates. The highlighted spectral family represents paenilipoheptin-like compounds (A). Subfamily 1 contains nodes that represent ions with masses corresponding to paenilipoheptin A (nodes highlighted with red circles). Exploring the mass features from subfamily 1 and 2 led to the discovery of paenilipoheptin A and B, respectively (B).

Subfamily 1 represents doubly charged ions with a mass range of 1080 to 1150 Da and contains 2 nodes of *m*/*z* 562.3133 (Figure 2B). However, the MS/MS fragmentation pattern of both of these nodes indicated differences in the amino acid sequences as compared to that reported for paenilipoheptin A.^12^ The differences lie in residues 2 and 4, which were previously annotated as Dab and Val, while our data suggested that they are Lys and Ala, respectively. The latter were confirmed by high-resolution MS/MS spectroscopy, which indeed revealed an ion corresponding to a Lys residue (*m*/*z* 129.1021), together with the fragment ions Ala–Trp (*m*/*z* 258.1240) and Ala–Phe (*m*/*z* 219.1122) (Figure S1). Further analysis of the *b* ions of the parent mass with *m*/*z* 562.3133 corroborated the lipopeptide sequence as FA(C_13_H_26_)–Ser–Lys–Trp–Ala–Phe–Tyr–Glu (Figure S1). In the original study, low-resolution MS was used to assign the structure of paenilipoheptin A,^12^ which might explain the discrepancy. To further verify that *Paenibacillus* sp. JJ-21 indeed produces paenilipoheptin A, we compared the compound to the one produced by *P. polymyxa* E681. For this, we cultured *P. polymyxa* E681 on TSA agar, employing the original extraction methods.^12^ Subsequent LC-MS/MS analysis of the crude extract from *P. polymyxa* E681 identified a paenilipoheptin with an *m*/*z* of 562.3142, which is identical to that of the paenilipoheptin produced by *Paenibacillus* sp. JJ-21. Additionally, comparative mirror plot analyses of the MS/MS fragmentation patterns from both strains confirmed that both of them produce paenilipoheptin A, with the sequence FA(C_13_H_26_)–Ser–Lys–Trp–Ala–Phe–Tyr–Glu (Figure S2).

The node with *m*/*z* 555.305 in subfamily 1 of the Lys-containing molecular family was linked to subfamily 2 via the node with *m*/*z* 523.8231. A mirror plot of the MS/MS spectra revealed that the two mass features shared numerous peaks in the low mass region (Figure S3). The common fragment ions correspond to the amino acids Lys, Tyr, and Trp, together with the dipeptide fragments Ser–Lys, Lys–Trp, and Trp–Ala. Interestingly, in addition to Lys, a fragment ion for Dab was detected in the MS/MS spectrum of the mass feature with *m*/*z* 523.8231. At the same time, no fragment ions for Glu or Phe were detected. These observations suggested that compounds from the second subfamily contained two positively charged amino acids, namely, Lys and Dab, and might be the products of the paenilipoheptin B BGC (Figure 1B). Upon further investigation of the *b* ions of the parent mass with *m*/*z* = 530.8307, we conclude that the lipopeptide sequence is FA(C_13_H_26_)–Ser–Lys–Trp–Ala–Leu–Tyr–Dab (Figure S4). *Paenibacillus* sp. JJ-21 and *Paenibacillus* sp. JJ-1722 stood out as they were found to produce substantial amounts of paenilipoheptins, and these strains were therefore chosen for larger scale fermentation in search of novel paenilipoheptin congeners.

*Paenibacillus* sp. JJ-21 and *Paenibacillus* sp. JJ-1722 were grown in 10 L Muller Hinton Broth (MHB) medium, and the biomass was collected by centrifugation. Specialized metabolites were extracted from the cells with isopropyl alcohol (IPA) supplemented with 0.1% (v/v) formic acid. The most abundant compounds, with *m*/*z* values of 562.3133 and 530.8307, were isolated from the extracts of *Paenibacillus* sp. JJ-21 and JJ-1722 through multiple rounds of high-performance liquid chromatography (HPLC) and were designated as paenilipoheptin A (**1**) and B (**2**), respectively. Compound **1** showed a molecular ion peak for an [M + H]^+^ ion having *m*/*z* 1123.6180 (calcd. for C_59_H_83_N_10_O_12_, 1123.6192) in the ESI-HRMS spectrum (Figure S5). Analysis of the 1D and 2D NMR spectra of compound **1** indicated the presence of seven amino acids: Ser, Lys, Trp, Ala, Phe, Tyr, Glu, and the β-amino fatty acyl chain (Table S1, Figure 3A, Figures S6–S12).

**Figure 3:**
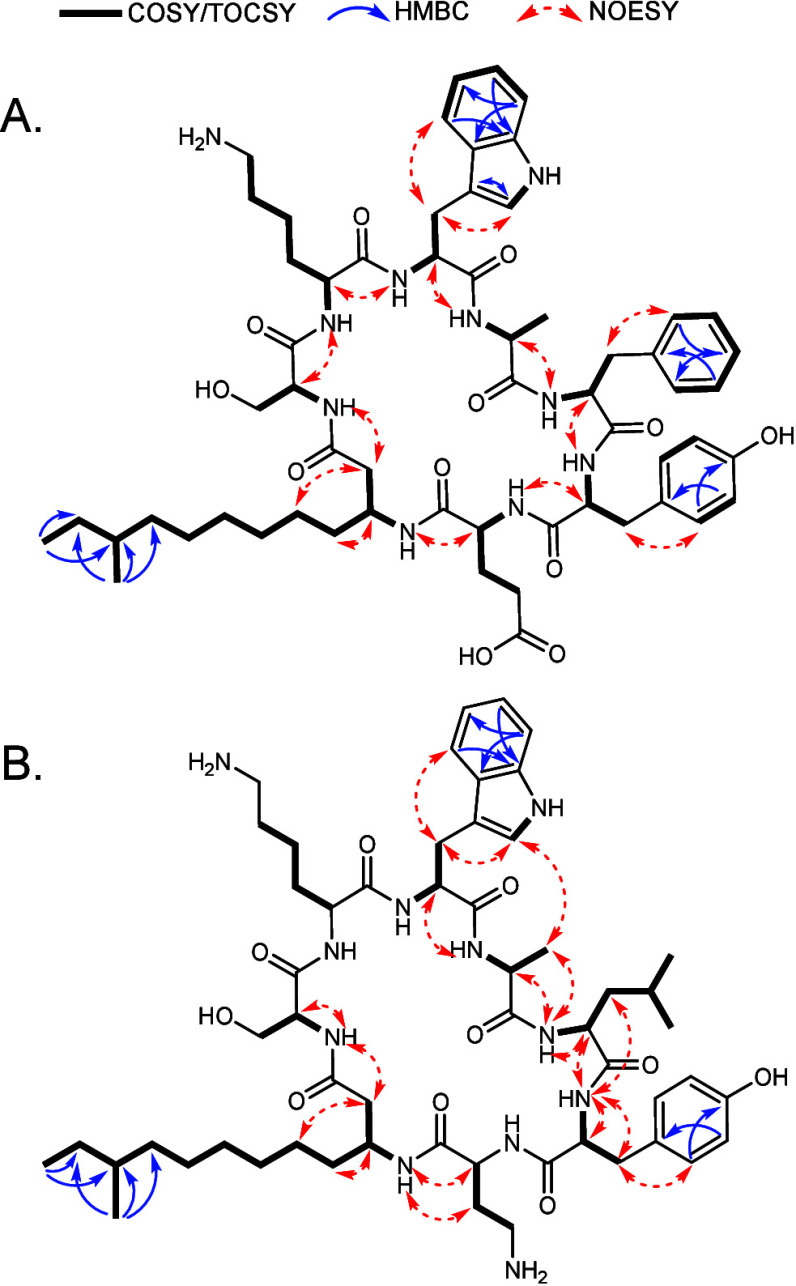
Correlations obtained by COSY, HMBC, and NOESY measurements in NMR of **1** (A) and **2** (B).

The β-amino fatty acid was identified as 3-amino-10-methyldodecanoic acid upon detailed NMR analysis. The NMR analysis thus confirmed that paenilipoheptin A contains Ala instead of Val at position 4 and Lys instead of Dab at position 2. For compound **2**, ESI-HRMS analysis revealed a molecular ion [M + H]^+^ with an *m*/*z* of 1060.6555 (calcd. for C_55_H_86_N_11_O_10_, 1060.6559) (Figure S13). The ^1^H NMR spectra of **1** and **2** were similar, indicating similar structures. Due to the low yield of **2**, the NMR signals were quite weak. Nevertheless, we could confirm the presence of Leu and Dab at positions 5 and 7, respectively, which differs from compound 1 that contains Phe and Glu residues, respectively, at these positions (Table S2, Figure 3B, Figures S14–S19).

The absolute configurations of the amino acids in compounds **1** and **2** were determined by Marfey’s analysis.^18^ This revealed Ser1, Phe5, and Tyr6 to be l-amino acids, whereas Lys2, Ala4, and Glu7 were found to be d-amino acids (Tables S3 and S4). The stereochemistry of Trp3 could not be established due to degradation of the Trp residue under the conditions used for generating the Marfey’s derivatives.

To characterize the paenilipoheptin BGCs, the genomes of *Paenibacillus* sp. JJ-21 and *Paenibacillus* sp. JJ-1722 were sequenced using the PacBio platform. Assembly of the PacBio reads with Falcon (version 1.8.1)^19^ resulted in single contigs of 6.2 and 6.1 Mb for *Paenibacillus* sp. JJ-21 (GenBank accession number: CP132974) and *Paenibacillus* sp. JJ-1722 (GenBank accession number: CP182500), respectively. An in silico analysis of the A domain substrate specificity and stereochemistry predictions for these BGCs were conducted with antiSMASH 7.1.0.^13^ The predicted amino acid composition of paenilipoheptin A of *Paenibacillus* sp. JJ-21 was identical with that of *P. polymyxa* E681, which was d-Ser–d-Dab–d-Trp–d-Val–l-Phe–l-Tyr–l-Glu (Table S5). The amino acid sequence predicted for paenilipoheptin B differed from paenilipoheptin A at positions 5 and 7, and was d-Ser–d-Dab–d-Trp–d-Val–l-Leu–l-Tyr–l-Dab.

Marfey’s analysis, MS/MS, and NMR studies revealed structural discrepancies with the previously predicted primary sequences for paenilipoheptin A (Table S3, S4). Specifically, Dab was predicted at position 2, but Lys was identified in the actual product. Similarly, Val was predicted at position 4, while in fact it is an Ala residue. Discrepancies between predicted and actual structures are likely due to database limitations in the bioinformatic tools used in making the previous structure predictions.^13^ Notably, the genomic predictions also differed from the actual paenilipoheptin structures in stereochemistry. For instance, in both paenilipoheptins A and B, l-Ser was found at position 1 instead of the previously predicted d-Ser. Amino acid sequences of E domains from modules 2–5 of paenilipoheptin BGCs of *Paenibacillus* sp. JJ-21 and *Paenibacillus* sp. JJ-1722 were also aligned to compare their active site motifs (HHxxxD).^21^ This revealed that the conserved active site motif HHxxxD of Ser epimerization domains is replaced with DPxxxD in both strains (Figure S20). Therefore, although epimerization (E) domains were detected in module 2 of the NRPSs encoded by these *Paenibacillus* genomes, we hypothesize that they are nonfunctional. Furthermore, d-Glu was detected at position 7 of paenilipoheptin A, rather than the predicted l-Glu, suggesting alternative mechanisms such as noncanonical epimerization or the involvement of an external enzyme. Additional studies are required to confirm these hypotheses.

Both paenilipoheptin A and B inhibited growth of *Bacillus subtilis* 168 with moderate bioactivity (minimal inhibitory concentration of 16–32 μg/mL), while no bioactivity against *Escherichia coli* ATCC 25922 was observed. A detailed investigation of the antimicrobial activity of paenilipoheptins is presented in our accompanying manuscript.^22^

In conclusion, genomic analysis and mass spectral networking revealed the high biosynthetic potential of *Paenibacillus* spp. as producers of NRPs with diverse chemistry. Bioinformatic analysis using BIG-SCAPE revealed that the paenilipoheptin GCFs contained BGCs associated not only with the previously described paenilipoheptin A but also with a new analogue here assigned as paenilipoheptin B. GNPS networking allowed us to identify the producers of these compounds for further purification and structure elucidation. This led to the structural revision of previously described paenilipoheptin A as well as the structure elucidation of the novel antibiotic paenilipoheptin B. These results further highlight the potential of *Paenibacillus* spp. for discovering NRPs and advancing the development of novel antibiotics.

## Data Availability

The data underlying this study are available in the published article, in its Supporting Information, and openly available at GNPS at https://gnps.ucsd.edu/ProteoSAFe/status.jsp?task=2f1e0ae1728142249ac4e841d5a72ef4, MassIVE Repository under accession number MSV000094386, NCBI under accession numbers CP132974 and CP182500.

## Supporting Information Available

General information, experimental procedures, NMR and MS/MS data for paenilipoheptin A and B isolated from natural source (PDF)
