# Supplementary material for: Exploring the Chemical Space of Paenibacillus NRPs and Discovery of Paenilipoheptin B
Source: Org Lett. 2025 Mar 14;27(12):2821–5. doi: 10.1021/acs.orglett.5c00231 (PMC11959601; doi:10.1021/acs.orglett.5c00231)
Supplement: Supplementary file 1 — ol5c00231_si_001.pdf [file ol5c00231_si_001.pdf]

Supporting Information for:

Exploring the Chemical Space of *Paenibacillus* NRPs and Discovery of Paenilipoheptin B

Nataliia V. Machushynets [a,‡], Vladyslav Lysenko [b,‡], Chao Du [a], Cornelis J. Slingerland [b], Somayah S. Elsayed [a], Mark R. Liles [c], Nathaniel I. Martin [b,*] and Gilles P. van Wezel [a,d,*]

[a] Molecular Biotechnology Group, Institute of Biology, Leiden University, Sylviusweg 72, 2333 BE, Leiden, The Netherlands

[b] Biological Chemistry Group, Institute of Biology, Leiden University, Sylviusweg 72, 2333 BE, Leiden, The Netherlands

[c] Department of Biological Sciences, Auburn University, 120 W Samford Av, AL 36849, Auburn, AL, USA

[d] Department of Microbial Ecology, Netherlands Institute of Ecology, Droevendaalsesteeg 10, 6708 PB, Wageningen, The Netherlands

‡ These authors contributed equally
* Corresponding authors: g.wezel@biology.leidenuniv.nl, n.i.martin@biology.leidenuniv.nl

List of Tables and Figures

Experimental Procedures……………………………………………………………………..3

Table S1. NMR data of paenilipoheptin A (**1**), measured in DMSO-d_6 at 298 K……………….8

Table S2. NMR data of paenilipoheptin B (**2**), measured in DMSO-d_6 at 298 K……………….9

Table S3. Retention times (t_R, min) of the FDAA derivatives for natural paenilipoheptin A and standard amino acids………………………………………………………………………..10

Table S4. Retention times (t_R, min) of the FDAA derivatives for natural paenilipoheptin B and standard amino acids……………………………………………………………………..…10

Table S5 Analysis of A-domain specificities of paenilipoheptin BGCs………………………....11

Figure S1. MS/MS spectrum of paenilipoheptin A (precursor ion $[M + 2H]^{2+}$ *m/z* 562.3133)..12

Figure S2. Comparison of the retention time and fragmentation patterns of paenilipoheptin from the extract of *Paenibacillus* sp. JJ-21 and *P. polymyxa* E681……………………………12

Figure S3. Direct MS/MS spectra comparison of the mass feature having *m/z* 555.305 (red) with the mass feature having *m/z* 523.8231 (blue)……………………………………………....13

Figure S4. MS/MS spectrum of paenilipoheptin B (precursor ion $[M + 2H]^{2+}$ *m/z* 530.8307)..13

Figure S5. HRMS spectrum of **1**……………………………………………………………..…14

Figure S6. ^{1}H NMR spectrum of **1** (850 MHz, in DMSO-d_6)……………………..………………14

Figure S7. ^{13}C{1H} APT spectrum of **1** (850 MHz, in DMSO-d_6)………………..……………..15

Figure S8. ^{1}H–^{1}H COSY spectrum of **1** (850 MHz, in DMSO-d_6)………………….……..……..15

Figure S9. ^{1}H-^{13}C HSQC spectrum of **1** (850 MHz, in DMSO-d_6)…………....………..……..16

Figure S10. ^{1}H–^{1}H TOCSY spectrum of **1** (850 MHz, in DMSO-d_6)....……….........…..…..16

Figure S11. ^{1}H–^{1}H NOESY spectrum of **1** (850 MHz, in DMSO-d_6)....………....………….…..17

Figure S12. ^{1}H-^{13}C HMBC spectrum of **1** (850 MHz, in DMSO-d_6)....………..…………..…..17

Figure S13. HRMS spectrum of **2**....……………………………………………………....……..18

Figure S14. ^{1}H NMR spectrum of **2** (850 MHz, in DMSO-d_6)....……………………...……..18

Figure S15. ^{1}H–^{1}H COSY spectrum of **2** (850 MHz, in DMSO-d_6)....………...…………….....19

Figure S16. ^{1}H-^{13}C HSQC spectrum of **2** (850 MHz, in DMSO-d_6)……....………...…..……..19

Figure S17. ^{1}H–^{1}H TOCSY spectrum of **2** (850 MHz, in DMSO-d_6)………....………...……..20

Figure S18. ^{1}H–^{1}H NOESY spectrum of **2** (850 MHz, in DMSO-d_6)………....………...……..20

Figure S19. ^{1}H-^{13}C HMBC spectrum of **2** (850 MHz, in DMSO-d_6)…………..……...……..21

Figure S20. Clustal2 multiple sequence alignment of the epimerization domains of the paenilipoheptin A and B BGCs from *Paenibacillus* sp. JJ-21 and *Paenibacillus* sp. JJ-1722, respectively……………………………………………………………………...………..……..21

Experimental procedures

General experimental procedures

NMR spectra were recorded on a Bruker Ascend 850 MHz NMR spectrometer (Bruker BioSpin GmbH). Data were analyzed using MestReNova 14 software (Mestrelab Research, Santiago de Compostela, Spain). High-performance liquid chromatography (HPLC) purification was performed on a Waters preparative HPLC system composed of a 1525 pump, a 2707 autosampler, a 2998 PDA detector, and a Water fraction collector III or BESTA-Technik preparative HPLC system equipped with an ECOM Flash UV detector. All solvents and chemicals were of HPLC or LC-MS grade, depending on the experiment. Structural assignments were made with additional information from NOESY, gCOSY, and gHSQC experiments.

Global genome mining of NRPSs

Genomes of all *Paenibacillus* spp. available from RefSeq (release 213)[1] were downloaded from the NCBI FTP site. All genomes with >400 contigs were considered as low-quality assemblies and were removed from the collection. All genomes were analyzed using AntiSMASH (version 6.0.1) to obtain BGC predictions. These predictions were then used as input into BiG-SCAPE (version 1.1.4),[2, 3] for the creation of a sequence similarity network, with distance matrix cutoff set to 0.25. The resulting full network was visualized by Cytoscape (3.9.1).[4]

Isolation and identification of bacterial strains

Paenibacillus strains were obtained from the Auburn University Plant-Associated Microbial strain collection. The similarity to the *P. polymyxa*, *P. peoriae* and *P. jamilae spp.* was calculated using 16S rRNA gene sequences of *Paenibacillus* spp. from Auburn University Plant-Associated Microbial strain collection obtained through PCR amplification with universal bacterial primers 27F and 1492R.[5] Each PCR amplicon was purified and used for Sanger sequencing. Subsequently, 16S rRNA gene sequences were assembled into consensus sequences and compared with reference sequences.

Growth of *Paenibacillus* spp. and natural product extraction

Cultivation and extraction of specialized metabolites was done following previously described method, with modifications.[6] Briefly, liquid pre-cultures of 25 *Paenibacillus* spp. from the Auburn University strain collection, namely, *Paenibacillus spp.* JJ-16, JJ-21, JJ-195, JJ-226, JJ-227, JJ-228, JJ-1580, JJ-1582, JJ-1603, JJ-1614, JJ-1638, JJ-1640, JJ-1652, JJ-1715, JJ-1720, JJ-1722, JJ-1724, JJ-333, JJ-845, JJ-1604, JJ-1650, JJ-1683, JJ-1729, JJ-1743, and JJ-1747 were used to inoculate as an inoculum for the 2.0 mL 96-deep well plate (Thermo Scientific, Nunc 2.0 mL DeepWell Plate) containing 600 µL TSA (TSA, Bacto Soybean-Casein

Digest Medium, 30 g/L). Plates were sealed with 96 Well-Cap Mats (Thermo Scientific, Nunc 96 Well-Cap Mats), and incubated at 30 °C for 72 h before extraction. The cultures were extracted twice with 300 µL 100% isopropanol acidified with 0.1% formic acid. The crude extracts were transferred into a pre-washed 96 well plate (Agilent Technologies, 96 well plates, 0.5 mL, polypropylene) and lyophilized to dryness. Dried samples were redissolved in 160 µL of methanol:water (1:1 v/v). *Paenibacillus polymyxa* E681 was cultivated on TSA, and metabolites were extracted following the protocol described in the previous study.[7]

Genome sequencing, assembly and annotation

Paenibacillus sp. JJ-21 and *Paenibacillus* sp. JJ-1722 were grown in TSB at 30 °C and 220 rpm for 24 h. DNA was extracted as described.[8] DNA quality was verified by agarose gel electrophoresis. PacBio sequencing and assembly was performed by Novogene (UK). Generally, libraries were prepared using SMRTbell template prep kit (PacBio, USA) according to manufacturer instructions. Sequencing was then performed using PacBio Sequel platform in continuous long reads mode. Assembly was done using Falcon (version 1.8.1).[9] BGCs in these genomes were annotated using AntiSMASH (version 7.1.0).[10]

Data-dependent LC-ESI-HRMS/MS

LC-MS/MS acquisition was performed using Shimadzu Nexera X2 ultra-high-performance liquid chromatography (UPLC) system, with an attached photodiode array detector (PDA), coupled to Shimadzu 9030 QTOF mass spectrometer, equipped with a standard electrospray ionization (ESI) source unit. A total of 2 µL was injected into a Waters Acquity HSS C_{18} column (1.8 µm, 100 Å, 2.1 × 100 mm) and data acquisition was performed as previously described.[11] Briefly, the gradient used was 5% B for 1 min, 5–85% B for 9 min, 85–100% B for 1 min, and 100% B for 4 min. All the samples were analyzed in positive polarity, using data-dependent acquisition mode. In this regard, full scan MS spectra (*m/z* 100–1700, scan rate 10 Hz, ID enabled) were followed by two data-dependent MS/MS spectra (*m/z* 100–1700, scan rate 10 Hz, ID disabled) for the two most intense ions per scan. The ions were fragmented using collision-induced dissociation (CID) with fixed collision energy (CE 20 eV) and excluded for 1 s before being re-selected for fragmentation.

MZmine 2 parameters

Prior to statistical analysis, mzXML files were imported into Mzmine 2.53[12] and processed as previously described [11]. Briefly, mass ion peaks were detected for MS^1 and MS^2 at a noise level of 2.0E2 and 0.0E0, respectively (positive polarity, mass detector: centroid), and their chromatograms were built using ADAP chromatogram builder. The detected peaks were smoothed, and the chromatograms were deconvoluted. The detected peaks were deisotoped (monotonic shape; maximum charge: 3; representative isotope: most intense). Peak lists from

different samples were aligned (weight for RT = weight for *m/z* = 50; compare the isotopic pattern with a minimum score of 50%). Only the features with MS/MS data were exported to a GNPS-FBMN. In addition, all features originating from the culture medium, as well as those with an m/z of less than 300, were removed.

Molecular network analysis and MassQL search

The resulting feature quantification table (CSV file) and MS/MS spectrum files (in mgf format) were uploaded to the GNPS webserver (http://gnps.ucsd.edu).[13] Briefly, the precursor ion mass tolerance was set to 0.005 Da and the MS/MS fragment ion tolerance to 0.05 Da. A molecular network was then created where edges were filtered to have a cosine score above 0.5 and more than 3 matched peaks. The molecular networks were visualized using Cytoscape software version 3.9.1[4] and displayed using an unweighted force-directed layout. The data are publicly accessible in the MassIVE repository (MSV000094386).

Up-scale fermentation, extraction, and isolation

Paenibacillus sp. JJ-21 and JJ-1722 were grown at 30 °C on tryptic soy agar (TSA) for 72 h and three colonies were inoculated into Tryptic Soy Broth (TSB) and incubated at 30 °C overnight. This inoculum (1%) was used to inoculate thirteen 2 L Erlenmeyer flasks containing 0.75 L of sterile Muller Hinton Broth (MHB) and fermented at 30 °C while shaking at 200 rpm for 72 h.

To extract the specialized metabolites produced by *Paenibacillus* sp. JJ-21 and JJ-1722, cells were collected by centrifugation (8000 rpm, 30 min, 4 °C) and washed with H_2O. Then, the cells were sonicated for 30 min and extracted with 100% isopropyl alcohol (IPA) supplemented with 0.1% (v/v) formic acid (FA) for 6 h. The crude extracts were collected, concentrated under reduced pressure and reconstituted in 50% ACN. The crude extract of *Paenibacillus* sp. JJ-21 was subjected to the preparative HPLC BESTA-Technik system (Dr. Maisch Reprosil Gold 120 C18 column (25 × 250 mm, 10 μm), buffer A (H_2O/MeCN/TFA = 95:5:0.1)/buffer B (H_2O/MeCN/TFA = 5:95:0.1) = 20-100, flow rate = 12.0 mL/min, l = 214 nm), t_R = 33.8 minutes to yield paenilipoheptin A (**1**, 1 mg, 0.9%). The crude extract of *Paenibacillus* sp. JJ-1722 was subjected to the Waters preparative HPLC system (SunFire C18 column (19 × 150 mm, 10 μm), buffer A (H_2O)/buffer B (MeCN) = 10-60, flow rate = 15.0 mL/min, l = 214 nm) resulting in three fractions. The third fraction was further purified (SunFire C18 column (10 × 250 mm, 5 μm), buffer A (H_2O/ACN = 75:25)/buffer B (H_2O/ACN = 65:35) = 25-35, flow rate = 3.0 mL/min, l = 214 nm) t_R = 13.48 minutes to yield paenilipoheptin B (**2**, 0.3 mg, 0.3%).

Paenilipoheptin A (**1**): white powder; ^{1}H NMR (850 MHz, DMSO-d_6) δ 12.01 (s, 1H), 10.81 (s, 1H), 9.22 (s, 1H), 8.42 (s, 1H), 8.24 (d, *J* = 4.4 Hz, 1H), 8.02 (s, 1H), 8.00 – 7.88 (m, 2H), 7.74 (d, *J* = 7.9 Hz, 1H), 7.70 (m, 1H), 7.69 – 7.60 (br s, 4H), 7.54 (d, *J* = 7.9 Hz, 1H), 7.33 (d, *J* = 8.0 Hz, 1H), 7.29 (d, *J* = 7.5 Hz, 2H), 7.21 (t, *J* = 7.5 Hz, 2H), 7.20 (br s, 1H), 7.16 – 7.12 (m,

2H), 7.08 – 7.02 (m, 3H), 6.98 (t, J = 7.4 Hz, 1H), 6.68 (d, J = 8.4 Hz, 2H), 4.80 (br s, 1H), 4.52 – 4.45 (m, 2H), 4.37 – 4.31 (m, 1H), 4.25 (q, J = 7.2, 6.8 Hz, 1H), 4.14 – 4.08 (m, 1H), 4.07 – 3.95 (m, 2H), 3.90 (s, 1H), 3.47 – 3.39 (m, 2H), 3.16 – 3.12 (m, 1H), 3.08 – 3.01 (m, 2H), 2.93 – 2.82 (m, 3H), 2.65 – 2.60 (m, 1H), 2.24 – 2.18 (m, 2H), 2.06 – 1.95 (m, 2H), 1.92 – 1.84 (m, 1H), 1.61 – 1.54 (m, 1H), 1.53 – 1.44 (m, 2H), 1.43 – 1.36 (m, 3H), 1.36 – 1.30 (m, 1H), 1.30 – 1.10 (m, 12H), 1.09 (d, J = 7.1 Hz, 3H), 1.08 – 0.96 (m, 3H), 0.83 – 0.77 (m, 6H). ^{13}C NMR (214 MHz, DMSO-d_6) δ 174.0, 172.1, 171.9, 171.5, 171.4, 170.8, 170.4, 170.0, 156.1, 138.1, 136.1, 130.1, 129.5, 128.0, 127.3, 127.0, 126.1, 123.5, 120.9, 118.32, 118.27, 118.1, 116.7, 115.0, 111.3, 110.5, 61.6, 5.9, 54.7, 54.4, 51.8, 49.1, 47.2, 41.7, 38.6, 37.2, 36.3, 36.0, 35.1, 33.7, 30.0, 29.9, 29.4, 28.92, 28.87, 27.17, 26.6, 26.5, 25.5, 22.3, 19.1, 17.9, 11.2; HRMS (ESI): *m/z* $[M+H]^+$ 1123.6180 (calcd. for $C_{59}H_{83}N_{10}O_{12}$, 1123.6192).

Paenilipoheptin B (**2**): white powder; 1H NMR (DMSO-*d6*, 850 MHz) δ 10.80 (s, 1H), 8.60 (s, 1H), 8.27 (s, 1H), 8.20 (s, 1H), 7.87 (s, 3H), 7.65 (s, 2H), 7.50 (d, J = 7.5 Hz, 1H), 7.32 (d, J = 7.5 Hz, 1H), 7.26 (s, 1H), 7.23 (s, 1H), 7.04 (t, J = 7.5 Hz, 1H), 6.96 (t, J = 7.5 Hz, 1H), 6.92 (d, J = 8.1 Hz, 2H), 6.56 (d, J = 8.1 Hz, 2H), 4.53 (m, 2H), 4.23 (m, 1H), 4.15 (m, 1H), 3.90 (m, 3H), 3.83 (s, 1H), 3.79 (d, J = 10.1 Hz, 1H), 3.65 (d, J = 10.1 Hz, 1H), 3.16 (m, 1H), 3.09 (m, 1H), 3.01 (m, 1H), 2.85 (d, J = 13.7 Hz, 1H), 2.65 (t, J = 13.0 Hz, 1H), 2.23 (s, 1H), 2.17 (d, J = 12.6 Hz, 1H), 1.65 (m, 2H), 1.59 (m, 1H), 1.51 (m, 2H), 1.45 (m, 3H), 1.35 (m, 3H), 1.23 (m, 16H), 0.82 (dd, J = 6.8, 2.5 Hz, 6H), 0.78 (d, J = 6.5 Hz, 3H), 0.68 (d, J = 6.5 Hz, 3H). ^{13}C chemical shifts (DMSO-*d6*, inferred from HSQC and HMBC spectra): δ 155.5, 135.8, 129.8, 127.7, 126.9, 123.5, 120.6, 118.1, 117.9, 114.3, 111.1, 62.0, 56.3, 55.3, 54.8, 52.8, 52.1, 51.7, 47.7, 47.1, 43.5, 40.0, 37.4, 37.3, 35.7, 34.9, 33.5, 33.2, 29.6, 29.5, 29.0, 28.7, 25.7, 23.8, 22.4, 22.2, 20.8, 18.8, 16.9, 10.9; HRMS (ESI): *m/z* $[M+H]^+$ 1060.6555 (calcd. for $C_{55}H_{86}N_{11}O_{10}$, 1060.6559).

Marfey`s analysis

The stereochemistry of chiral centers present at α carbons were assigned by applying derivatization methods coupled with chromatographic analysis. The advanced Marfey's method using L-FDAA (1-fluoro-2-4-dinitrophenyl-5-L-alanine amide) established the absolute configurations of amino acids.[14] The general method for Marfey`s analysis was conducted as described).[15] Briefly, a sample of peptide (30 µg) in 6M HCl (100 µL) was heated to 100 °C in a sealed vial for 8−12 h using heating block, after which the hydrolysate was concentrated to dryness at 40 °C under a stream of dry N2. The hydrolysate was then treated with 1 M $NaHCO_3$ (20 µL) and L-FDAA (1% solution in acetone, 40 µL) at 40 °C for 1 h, after which the reaction was neutralized with 1 M HCl (20 µL). An aliquot of the analyte was diluted 50 times with H_2O/ACN (1:1) and injected (2 µL) into an HRMS instrument following the standard protocol of

the analysis. The analyte amino acid content was assessed by comparison to authentic standards. The authentic standards were prepared via a similar procedure.

Table S1. NMR data of paenilipoheptin A (**1**), measured in DMSO-d_6 at 298 K*.

	Residue	NH	Hα (Cα, type)	Hβ (Cβ, type)	Other
**1**	**Fatty acid**	7.20	2.21 (41.7, CH_2)	4.00 (47.2, CH)	1 C: (170.4)<br>4 CH_2: 1.39, 1.33 (35.11)<br>5-8 CH_2: 1.05-1.25 (25.5)<br>9 CH_2: 1.20, 1.00 (36.30)<br>10 CH: 1.25 (33.7)<br>11 CH_2: 1.26, 1.07 (29.0)<br>12 CH_3: 0.81 (11.18)<br>13 CH_3: 0.79 (19.07)
**2**	**1-Ser**	7.74	4.48 (54.4, CH)	3.44, 3.41 (61.63, CH_2)	1C: ND<br>OH: 4.80
**3**	**2-Lys**	8.42	3.90 (54.7, CH)	1.49 (30.0, CH_2)	1C: ND<br>4 CH_2: 1.16, 1.05 (22.3)<br>5 CH_2: 1.39 (26.6)<br>6 CH_2: 2.62 (38.6)<br>NH_2: ND
**4**	**3-Trp**	8.02	4.48 (54.4, CH)	3.13, 3.06 (27.17, CH_2)	1 C: ND<br>4 C: (110.5)<br>5 CH: 7.14 (123.5)<br>6 C: (136.1)<br>7 CH: 7.33 (111.3)<br>8 CH: 7.06 (120.9)<br>9 CH: 6.98 (118.27)<br>10 CH: 7.54 (118.3)<br>11 C: (127.2)<br>NH: 10.81
**5**	**4-Ala**	7.70	4.03 (49.14, CH)	1.09 (17.85, CH_3)	1 C: (172.1)
**6**	**5-Phe**	7.92	4.33 (54.7, CH)	3.05, 2.90 (37.2, CH_2)	1 C: ND<br>4 C: 138.1<br>5 & 9 CH: 7.29 (129.5)<br>6 & 8 CH: 7.21 (128.0)<br>7 CH: 7.14 (126.1)
**7**	**6-Tyr**	8.24	4.25 (55.9, CH)	2.89, 2.85 (36.0, CH_2)	1 C: (170.75)<br>4 C: (127.05)<br>5 & 9 CH: 7.05 (130.1)<br>6 & 8 CH: 6.67 (115.0)<br>7 C: (156.3)<br>OH: 9.22
**8**	**7-Glu**	7.95	4.11 (51.8, CH)	1.88, 1.57 (26.6, CH_2)	1 C: ND<br>4 CH_2: 2.00 (29.9)<br>5 C: (174.0)

* ^{1}H 850 MHz and ^{13}C 212.5 MHz

ND: not determined under these experimental conditions.

Table S2. NMR data of paenilipoheptin B (**2**), measured in DMSO-d_6 at 298 K*.

	Residue	NH	H_α(C_α, type)	H_β(C_β, type)	Other
**1**	**Fatty acid**	8.20	2.23, 2.17 (43.5, CH_2)	3.87 (47.1, CH)	4 CH_2: 1.49 (34.9)<br>5-8 CH_2: 1.23 (28.7)<br>9 CH_2: 1.24 (35.7)<br>10 CH: 1.28 (33.5)<br>11 CH_2: 1.26, 1.07 (29.0)<br>12 CH_3: 0.83 (10.9)<br>13 CH_3: 0.82 (18.8)
**2**	**1-Ser**	7.58	4.53 (55.3, CH)	3.79, 3.65 (62.0, CH_2)	
**3**	**2-Lys**	ND	3.83 (56.3, CH)	1.59, 1.53 (29.5, CH_2)	4 CH_2: 1.21, 1.12 (22.2)<br>5 CH_2: 1.34 (29.6)<br>6 CH_2: ND<br>NH_2: ND
**4**	**3-Trp**	8.60	4.23 (54.8, CH)	3.16, 3.09 (25.7, CH_2)	4 C: ND<br>5 CH: 7.26, (123.5)<br>6 NH: 10.8<br>7 C: 135.8<br>8 CH: 7.32 (111.1)<br>9 CH: 7.04 (120.6)<br>10 CH: 6.96 (118.1)<br>11 CH: 7.50 (117.9)<br>12 C: 126.9
**5**	**4-Ala**	7.16	4.15 (47.7, CH)	1.12 (16.9, CH_3)	
**6**	**5-Leu**	7.23	3.89 (52.1, CH)	1.35, 1.05 (40.0, CH_2)	4 CH: 1.45 (23.8)<br>5 CH_3: 0.68 (20.8)<br>6 CH_3: 0.78 (22.4)
**7**	**6-Tyr**	7.65	4.53 (51.7, CH)	2.85, 2.65 (37.3, CH_2)	4 C: 127.7<br>5 & 9 CH: 6.92 (129.8)<br>6 & 8 CH: 6.56 (114.3)<br>7 C: 155.5
**8**	**7-Dab**	ND	3.91 (52.8, CH)	1.65 (33.2, CH_2)	4 CH_2: 2.56 (37.4)<br>NH_2: ND

* ^{1}H 850 MHz and ^{13}C chemical shifts inferred from HSQC and HMBC spectra

ND: not determined under these experimental conditions (and also all carbonyl carbons).

Table S3. Retention times (t_R, min) of the FDAA derivatives for natural paenilipoheptin A and standard amino acids.

	$[M+H]^+$	t_R, min			
		L-AA (standard)	D-AA (standard)	Paen A	Stereochemical assignment
**Ser**	358.0994	4.66	4.72	4.66	**L**
**Lys**	651.2121	6.50	6.67	6.68	**D**
**Trp**	457.1466	6.37	6.63	-	**NA**
**Ala**	342.1044	5.28	5.62	5.62	**D**
**Phe**	418.1357	6.51	6.90	6.52	**L**
**Tyr***	686.1801	7.09	7.48	7.09	**L**
**Glu**	400.1099	4.97	5.12	5.12	**D**
**FA**	482.2609	NA	NA	10.17	**L**

* Product of double-addition of Marfey`s reagent

NA: not available

Table S4. Retention times (t_R, min) of the FDAA derivatives for natural paenilipoheptin B and standard amino acids.

	$[M+H]^+$	t_R, min			
		L-AA (standard)	D-AA (standard)	Paen B	Stereochemical assignment
**Ser**	358.0994	4.66	4.72	4.66	**L**
**Lys**	651.2121	6.50	6.67	6.68	**D**
**Trp**	457.1466	6.37	6.63	-	**NA**
**Ala**	342.1044	5.28	5.62	5.62	**D**
**Leu**	384.1514	6.45/6.55	6.98/7.04	6.45/6.55	**L**
**Tyr***	686.1801	7.09	7.48	7.09	**L**
**Dab***	623.1804	6.23	6.38	6.23	**L**
**FA**	482.2609	NA	NA	10.17	**L**

* Product of double-addition of Marfey`s reagent

NA: not available

Table S5 Analysis of A-domain specificities of paenilipoheptin BGCs.

**Domain**	**Residues in the binding pocket**			**Amino acid prediction**			**Amino acid detected**		
	PaenA BGC of *P. polymyxa* E681	PaenA BGC of *Paenibacillus* sp. JJ-21	PaenB BGC of *Paenibacillus* sp. JJ-1722	PaenA BGC of *P. polymyxa* E681	PaenA BGC of *Paenibacillus* sp. JJ-21	PaenB BGC of *Paenibacillus* sp. JJ-1722	PaenA of *P. polymyxa* E681	PaenA *Paenibacillus* sp. JJ-21	PaenB *Paenibacillus* sp. JJ-1722
**M2**	DVWHFSLVDK	DVWHFSLVDK	DVWHFSLVDK	Ser	Ser	Ser	Ser	Ser	Ser
**M3**	DVCETGTIEK	DVCETGTIEK	DVCETGTIEK	Dab	Dab	Dab	Lys	Lys	Lys
**M4**	DAWAFAGVAK	DAWAFAGVAK	DAWAFAGVAK	Trp	Trp	Trp	Trp	Trp	Trp
**M5**	DVFWLGGTFK	DVFWMGGTFK	DVFWLGGTFK	Val	Val	Val	Ala	Ala	Ala
**M6**	DAWTFAAIAK	DAWTFAAIAK	DAWIFGAITK	Phe	Phe	Leu	Phe	Phe	Leu
**M7**	DTSTLAAVAK	DTSTLAAVAK	DTSTLAAVAK	Tyr	Tyr	Tyr	Tyr	Tyr	Tyr
**M8**	DAKDIGVVDK	DAKDIGVVDK	DVGEISSIDK	Glu	Glu	Dab	Glu	Glu	Dab

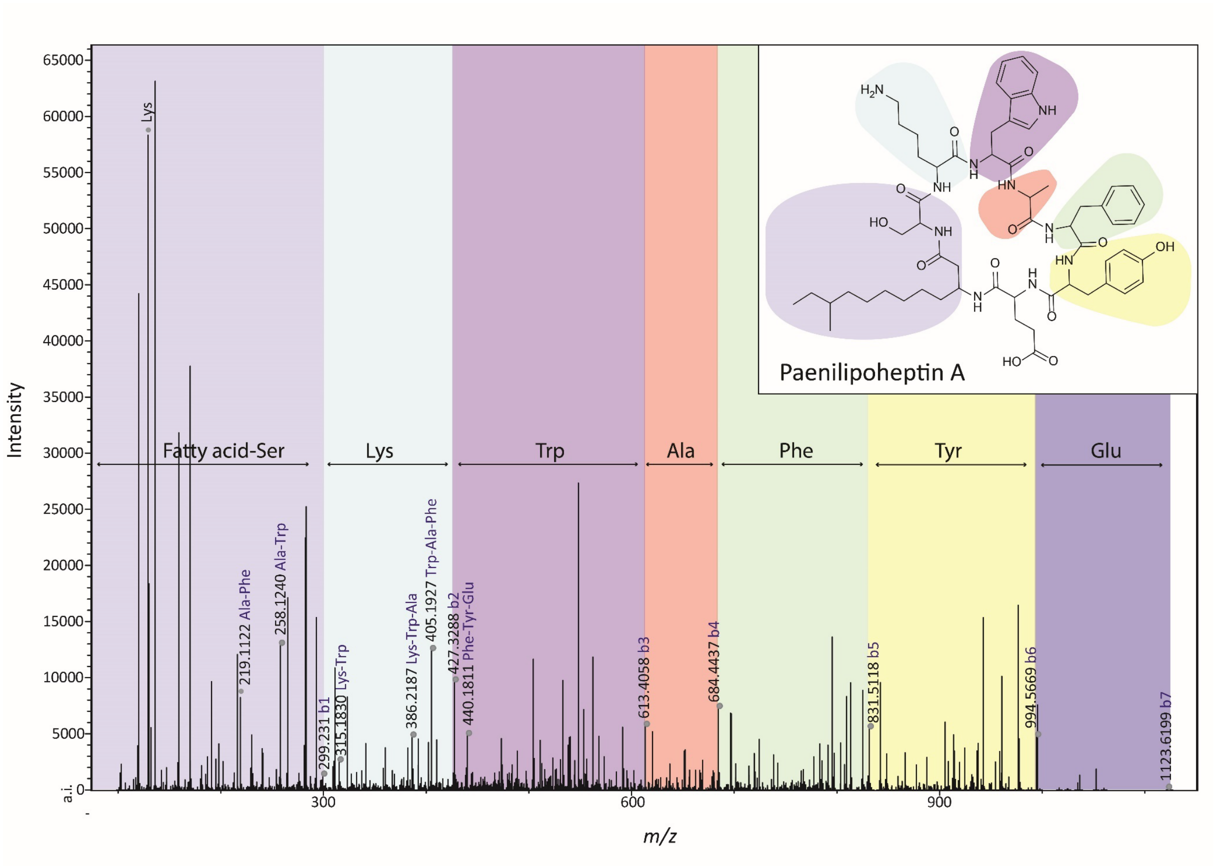

Figure S1. MS/MS spectrum of paenilipoheptin A (precursor ion $[M+2H]^{2+}$ *m/z* 562.3133). The assignment of the sequence of amino acid residues is based on the mass differences between the consecutive *b* ions.

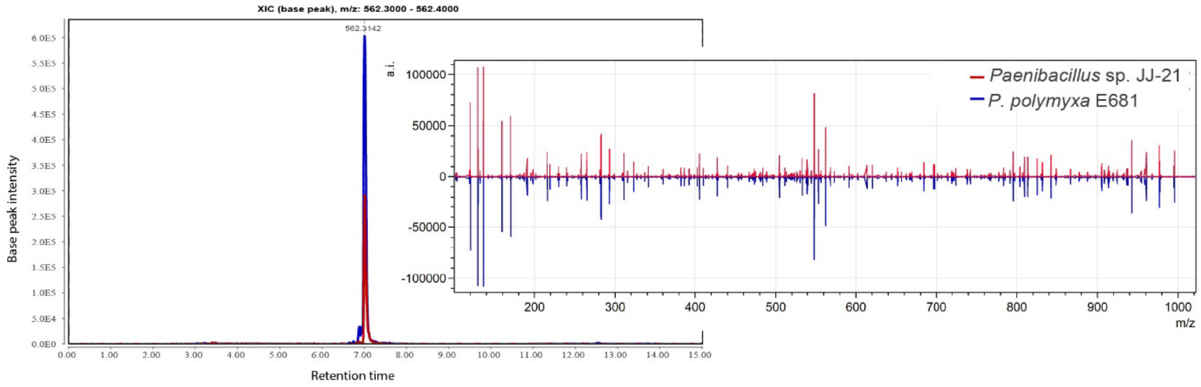

Figure S2. Comparison of the retention time and fragmentation patterns of paenilipoheptin from the extract of *Paenibacillus* sp. JJ-21 and *P. polymyxa* E681.

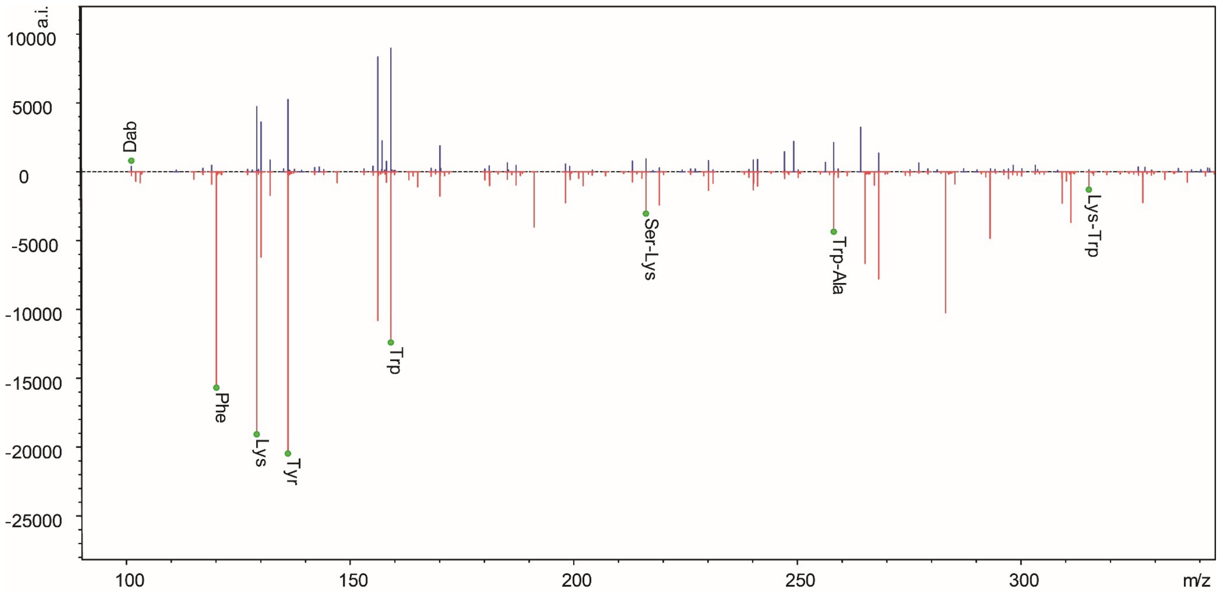

Figure S3. Direct MS/MS spectra comparison of the mass feature having *m/z* of 555.305 (red) with the mass feature having *m/z* of 523.8231 (blue).

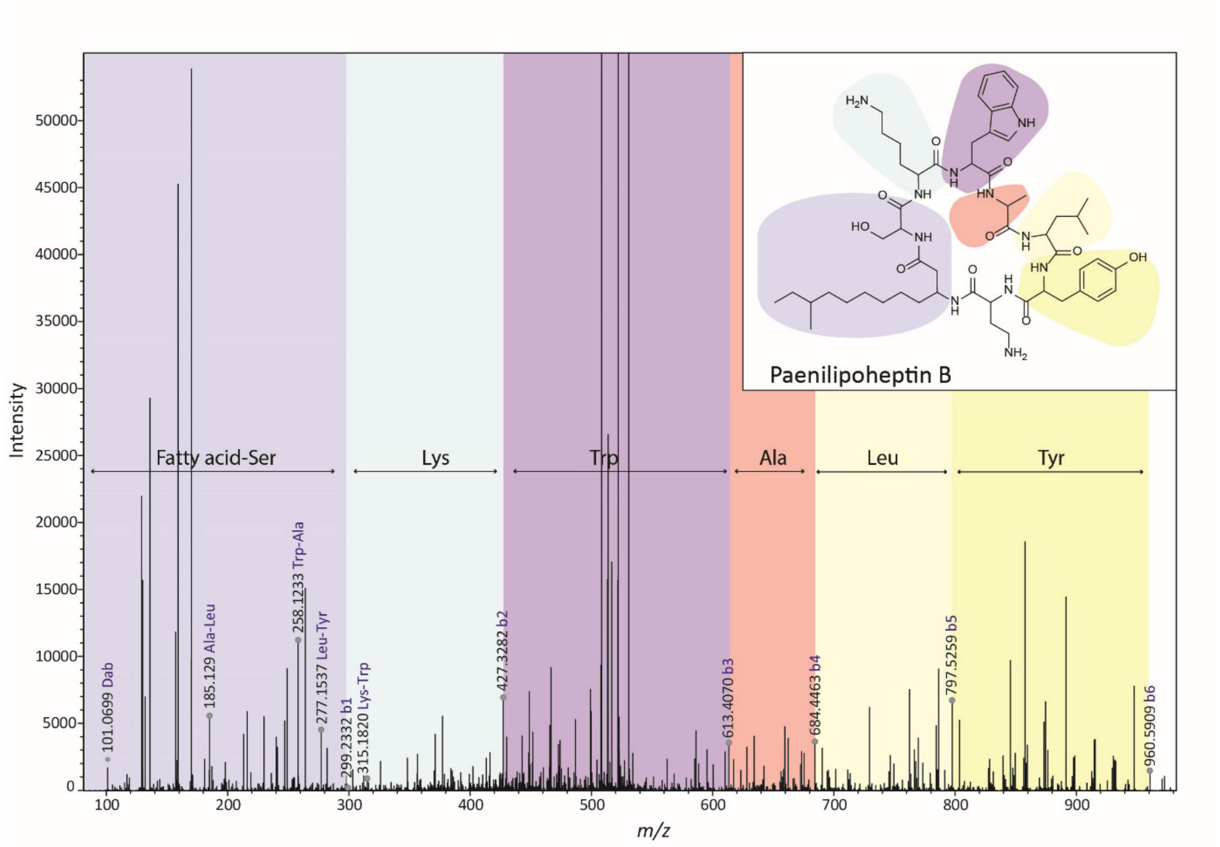

Figure S4. MS/MS spectrum of paenilipoheptin B (precursor ion $[M+2H]^{2+}$ *m/z* 530.8307). The assignment of the sequence of amino acid residues is based on the mass differences between the consecutive *b* ions.

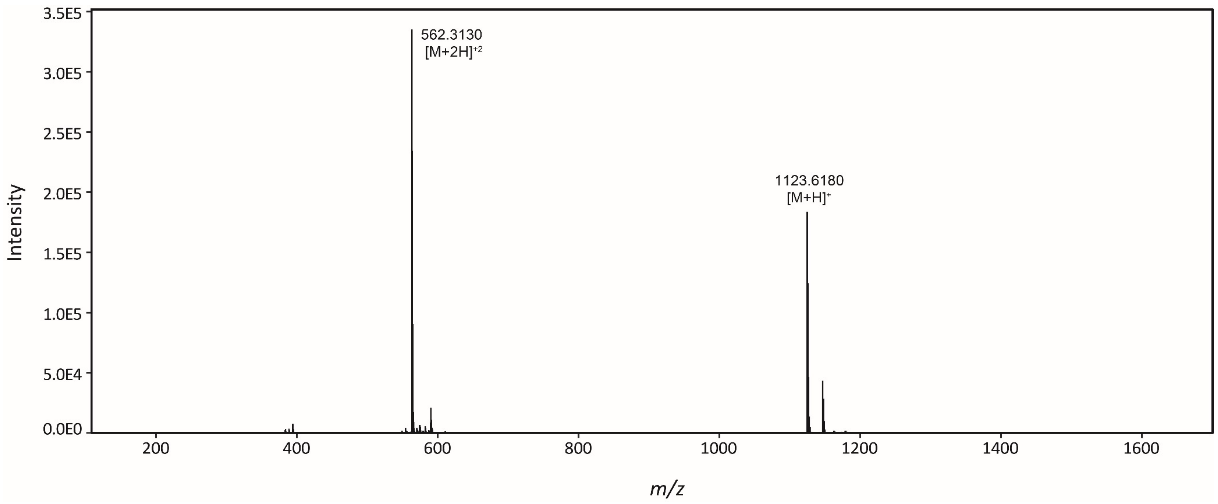

Figure S5. HRMS spectrum of **1**.

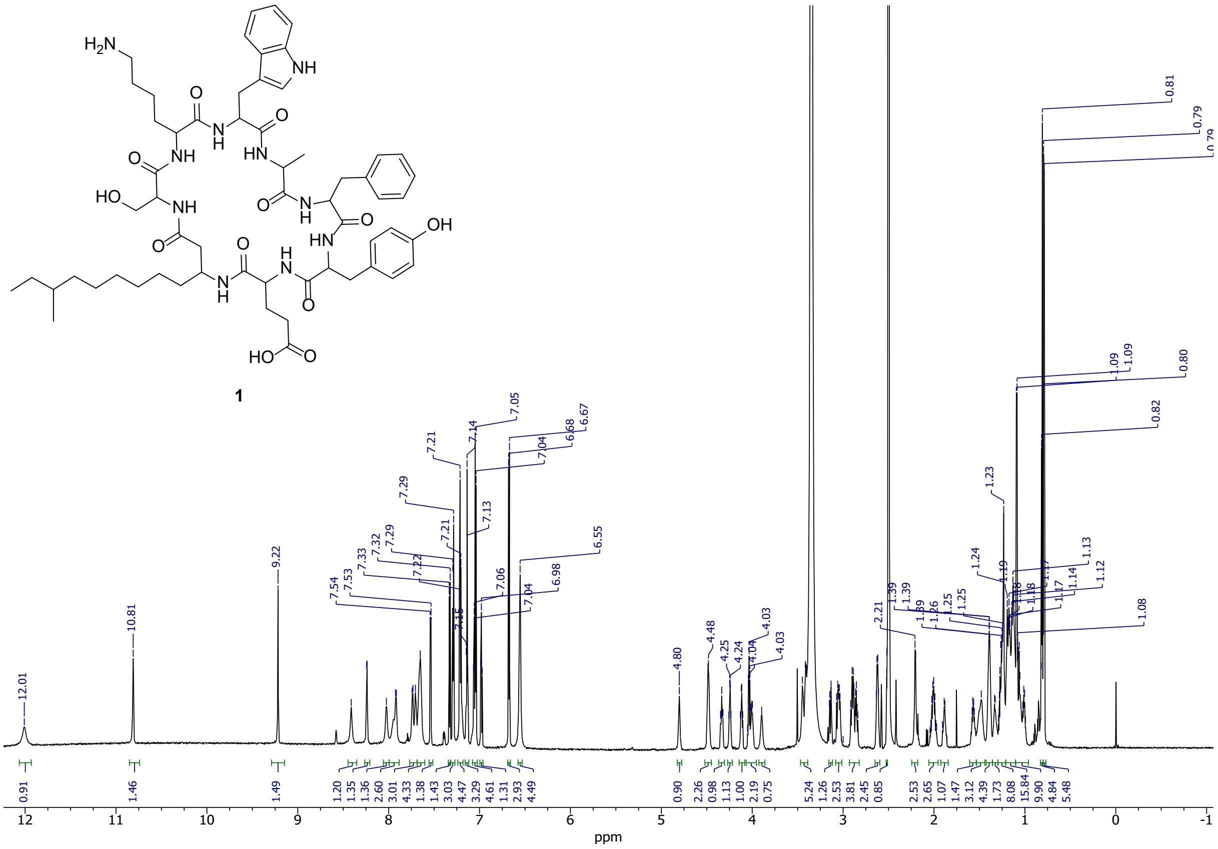

Figure S6. ^{1}H NMR spectrum of **1** (850 MHz, in DMSO-d_6).

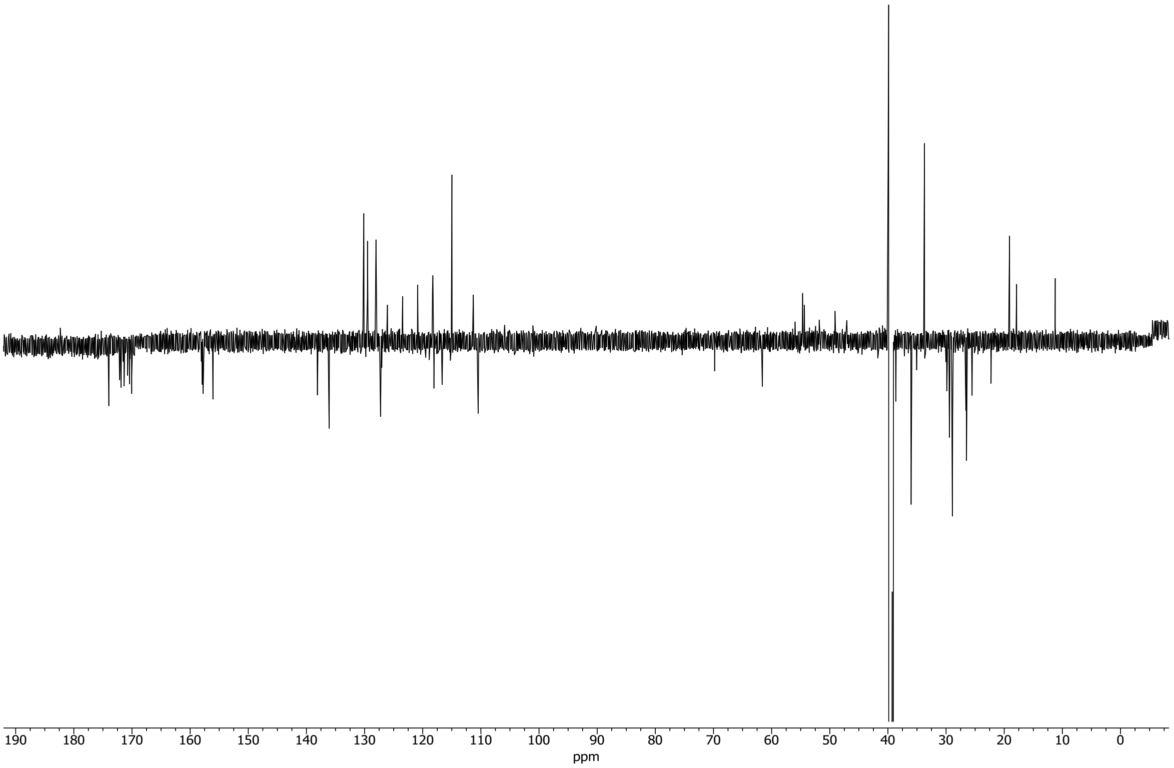

Figure S7. $^{13}C\{1H\}$ APT spectrum of **1** (850 MHz, in DMSO-d_6).

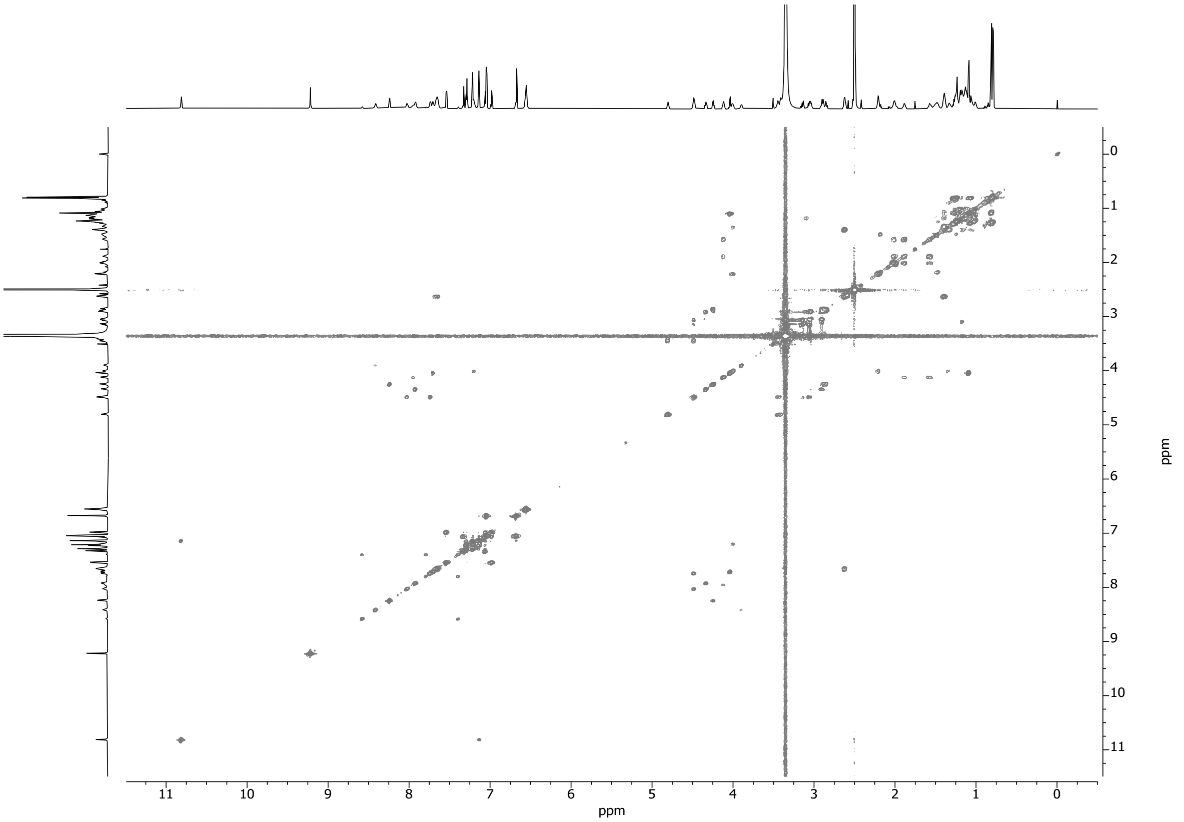

Figure S8. ^1H–^1H COSY spectrum of **1** (850 MHz, in DMSO-d_6).

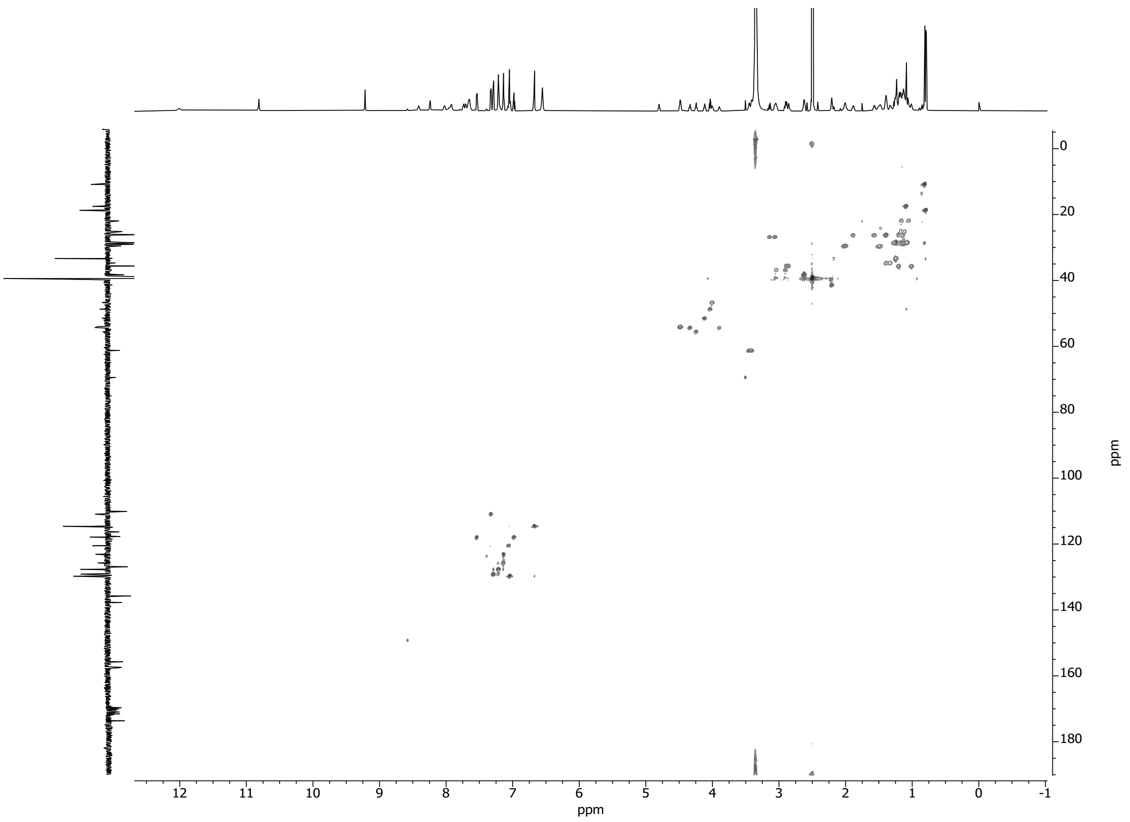

Figure S9. ^{1}H-^{13}C HSQC spectrum of **1** (850 MHz, in DMSO-d_6).

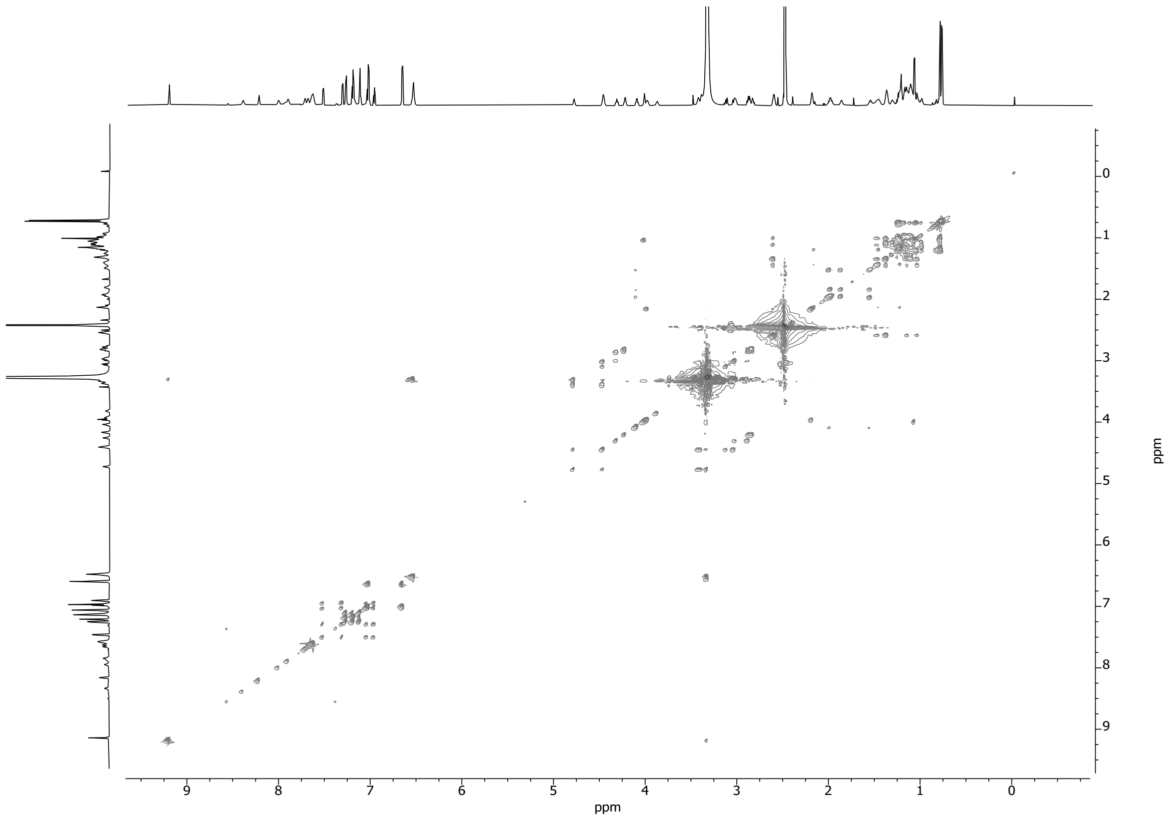

Figure S10. ^{1}H–^{1}H TOCSY spectrum of **1** (850 MHz, in DMSO-d_6).

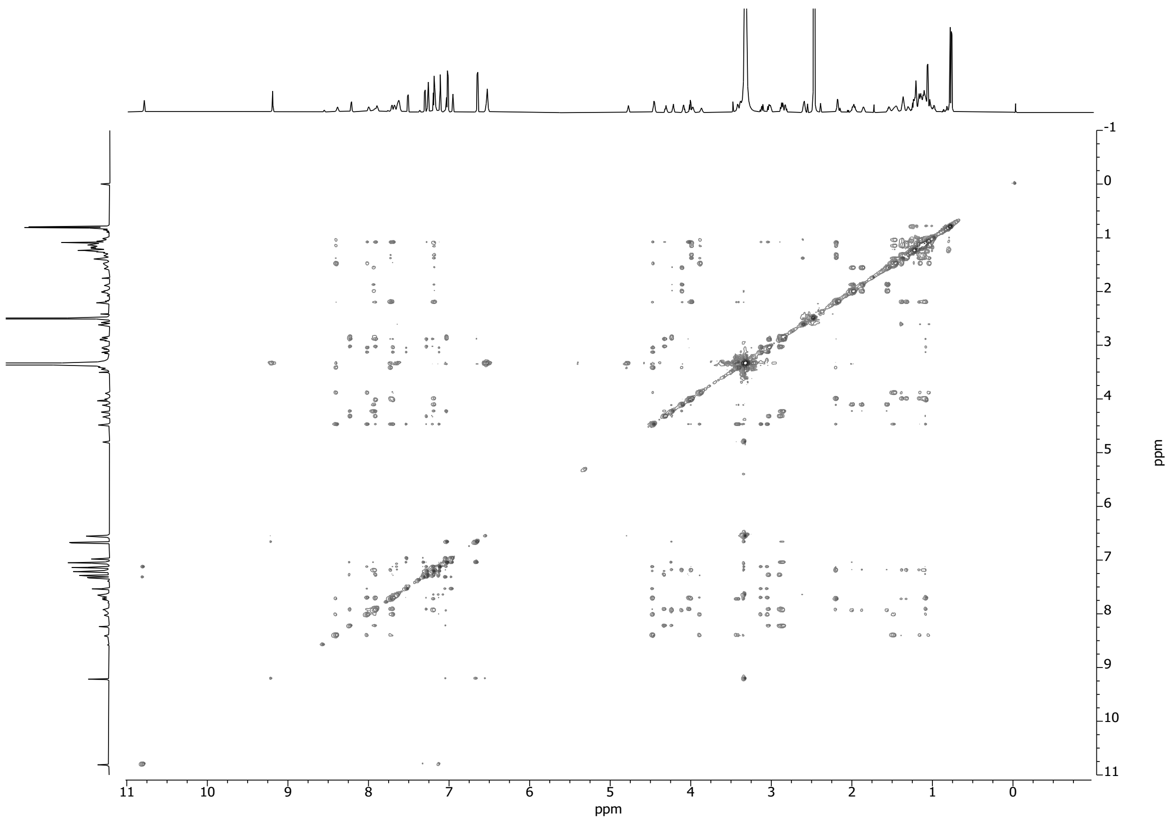

Figure S11. ^{1}H-^{1}H NOESY spectrum of **1** (850 MHz, in DMSO-d_6).

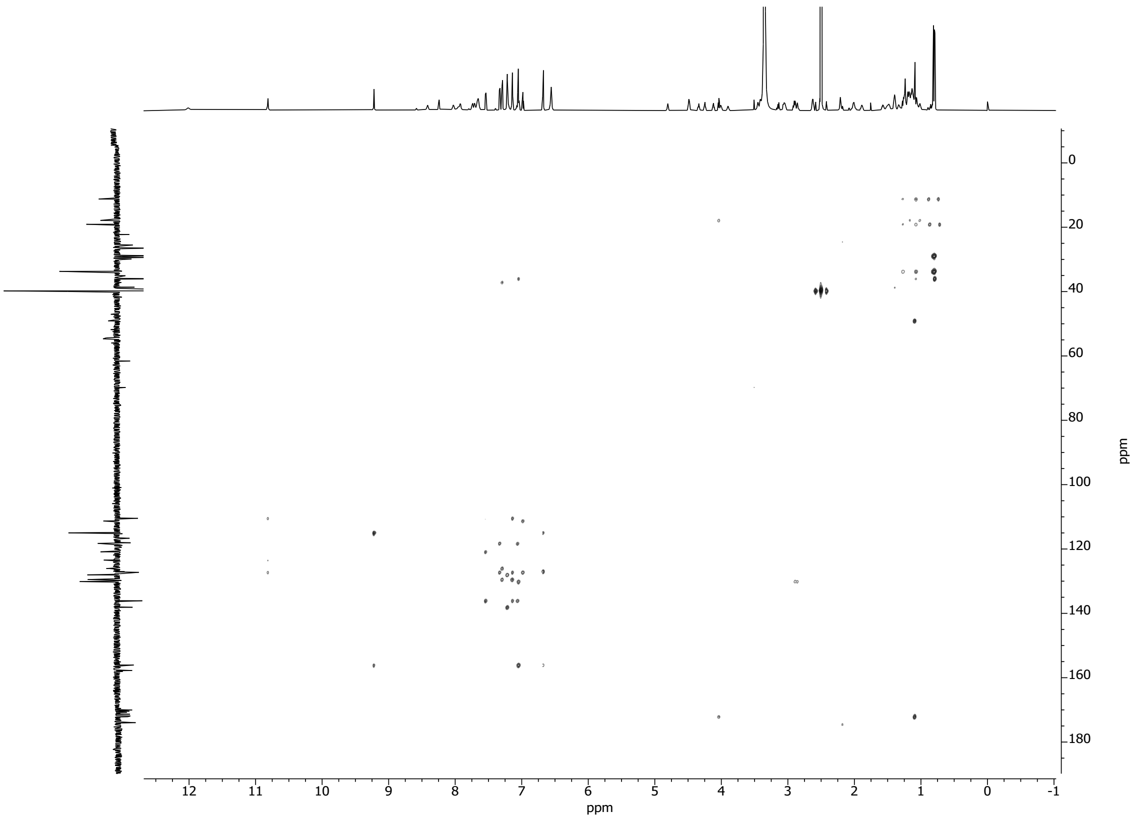

Figure S12. ^{1}H-^{13}C HMBC spectrum of **1** (850 MHz, in DMSO-d_6).

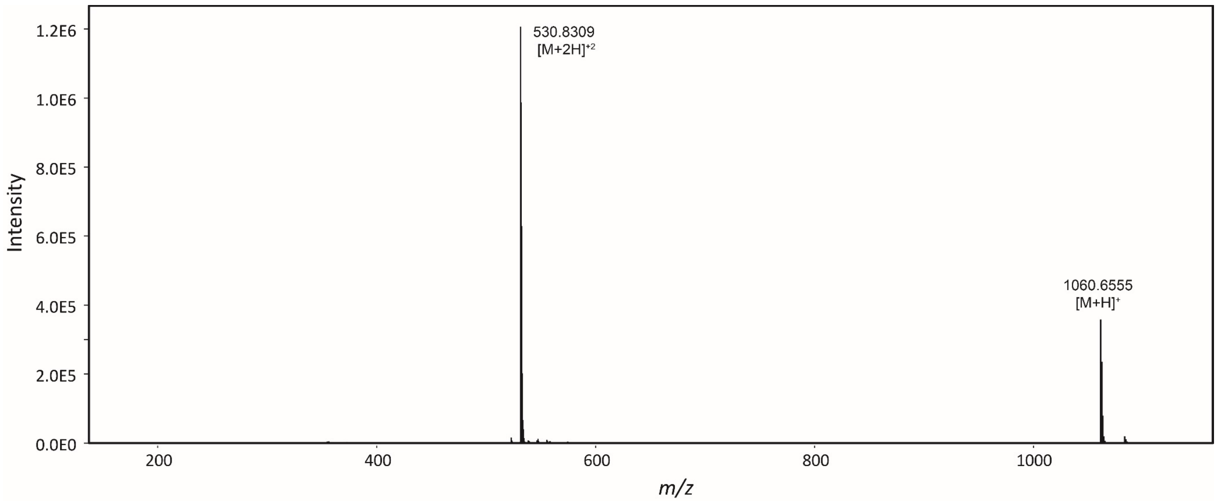

Figure S13. HRMS spectrum of **2**.

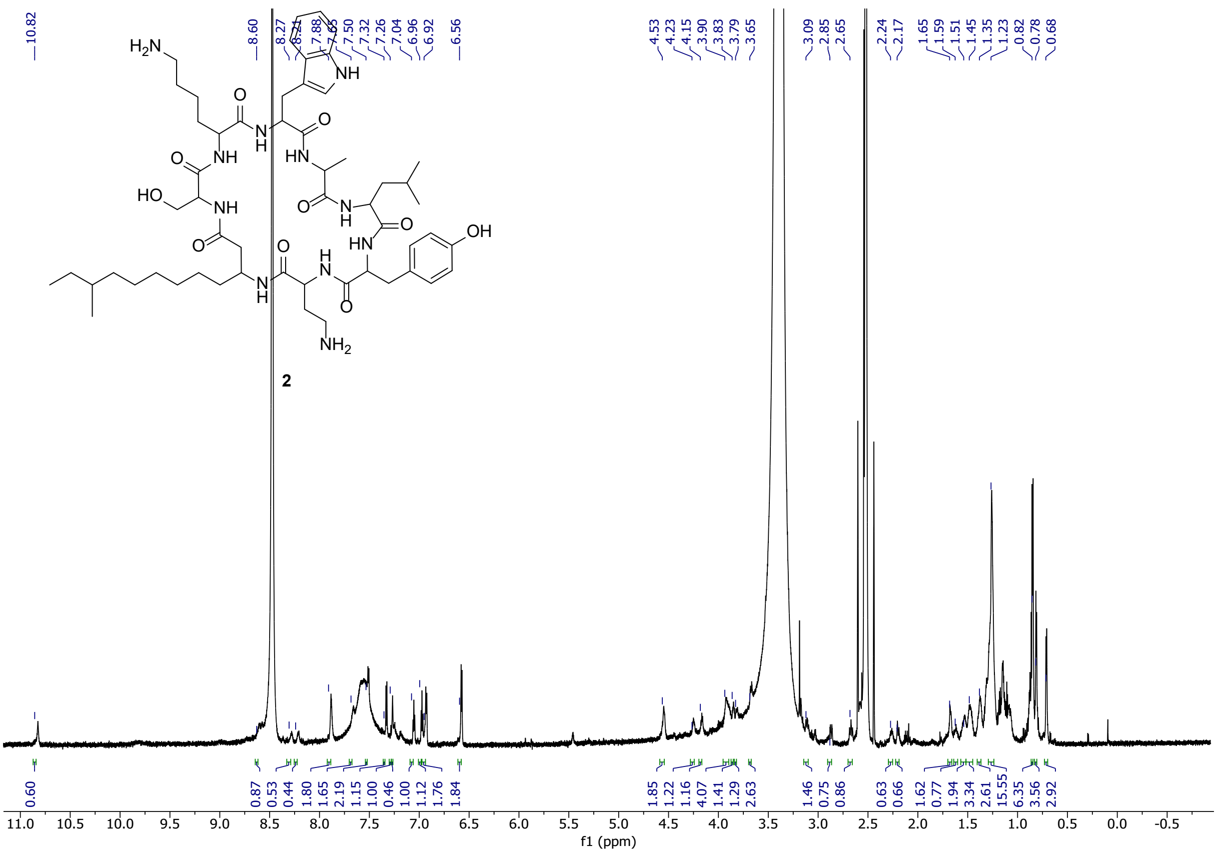

Figure S14. ^{1}H NMR spectrum of **2** (850 MHz, in DMSO-d_6).

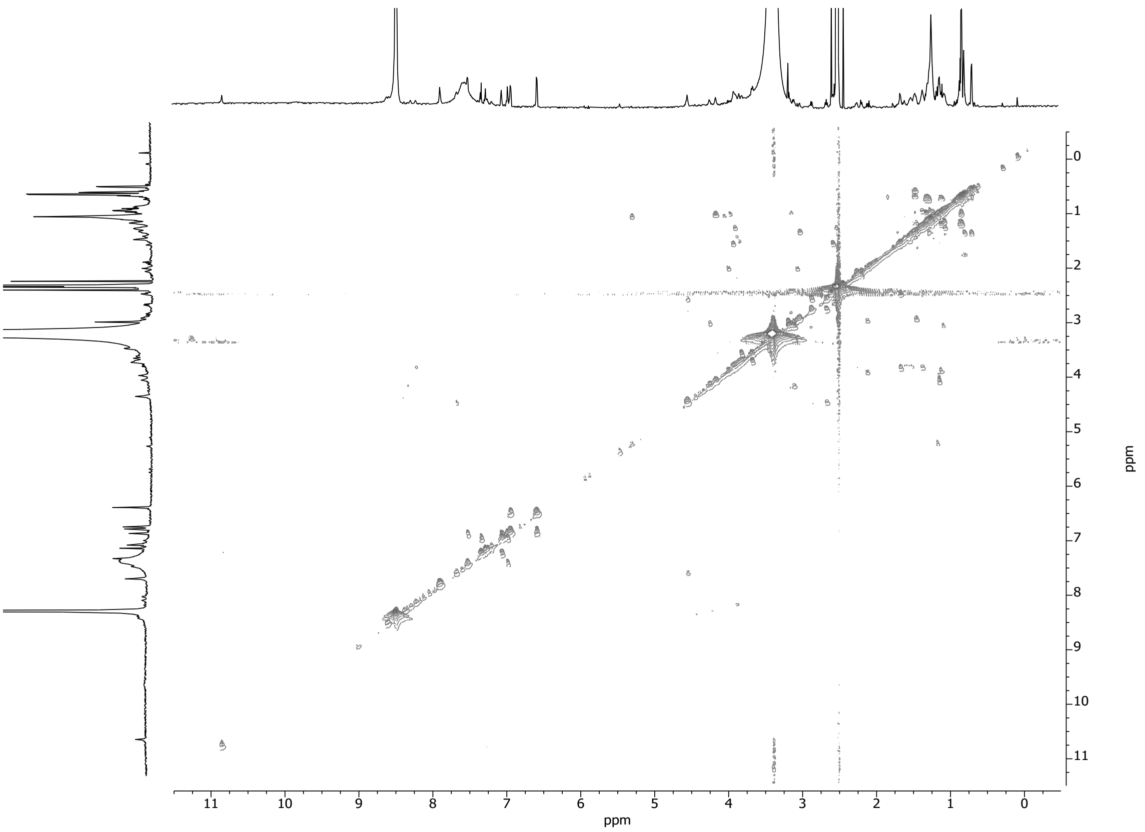

Figure S15. ^{1}H–^{1}H COSY spectrum of **2** (850 MHz, in DMSO-d_6).

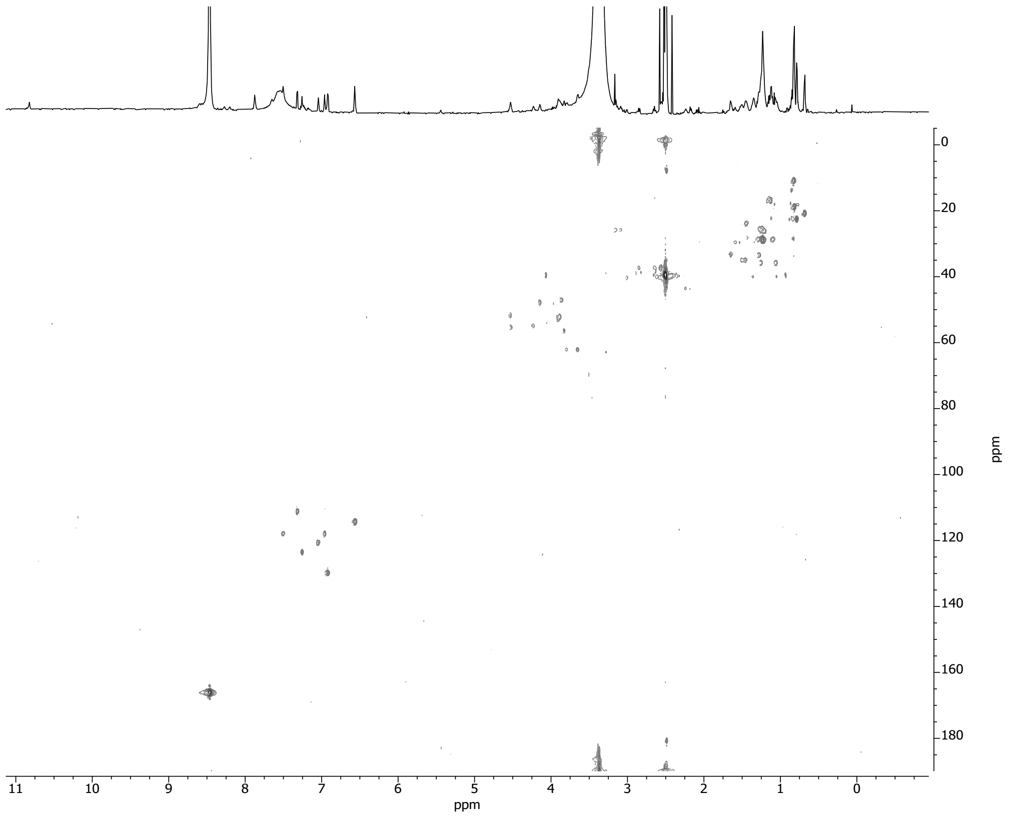

Figure S16. ^{1}H-^{13}C HSQC spectrum of **2** (850 MHz, in DMSO-d_6).

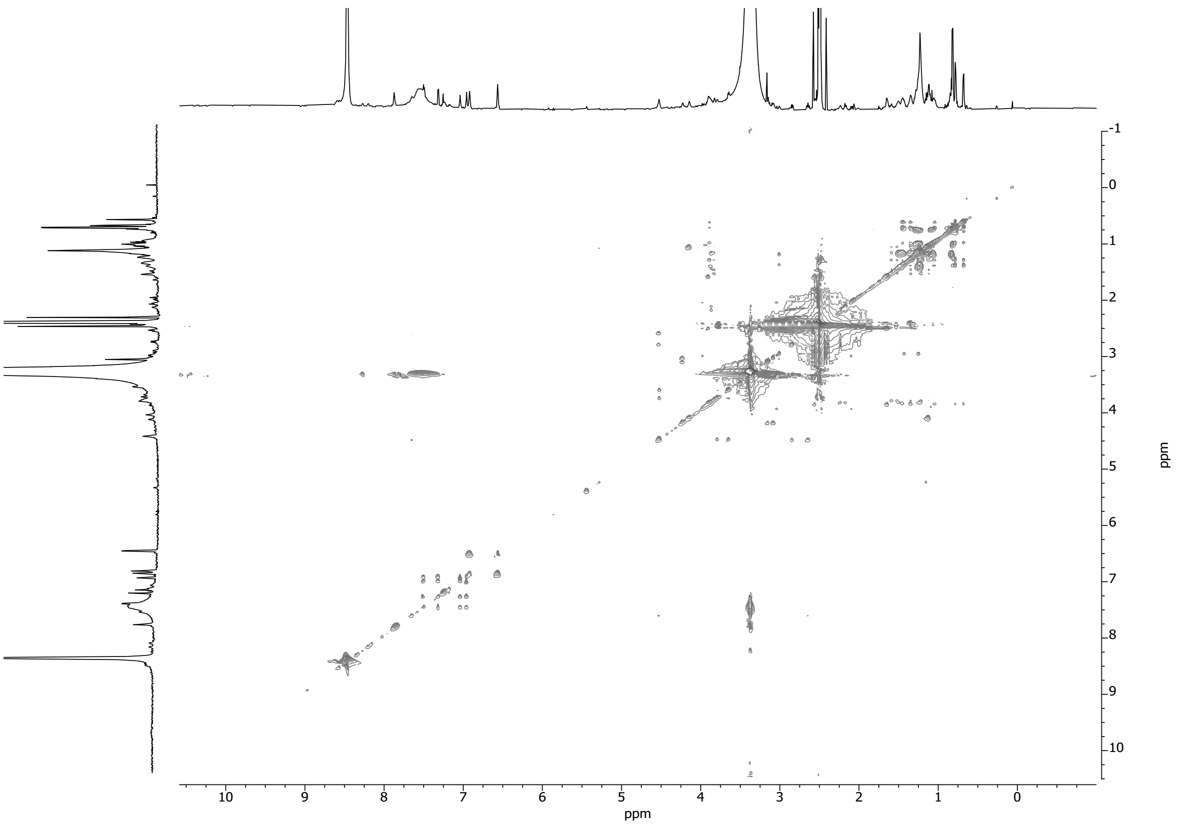

Figure S17. ¹H–¹H TOCSY spectrum of **2** (850 MHz, in DMSO-*d₆*).

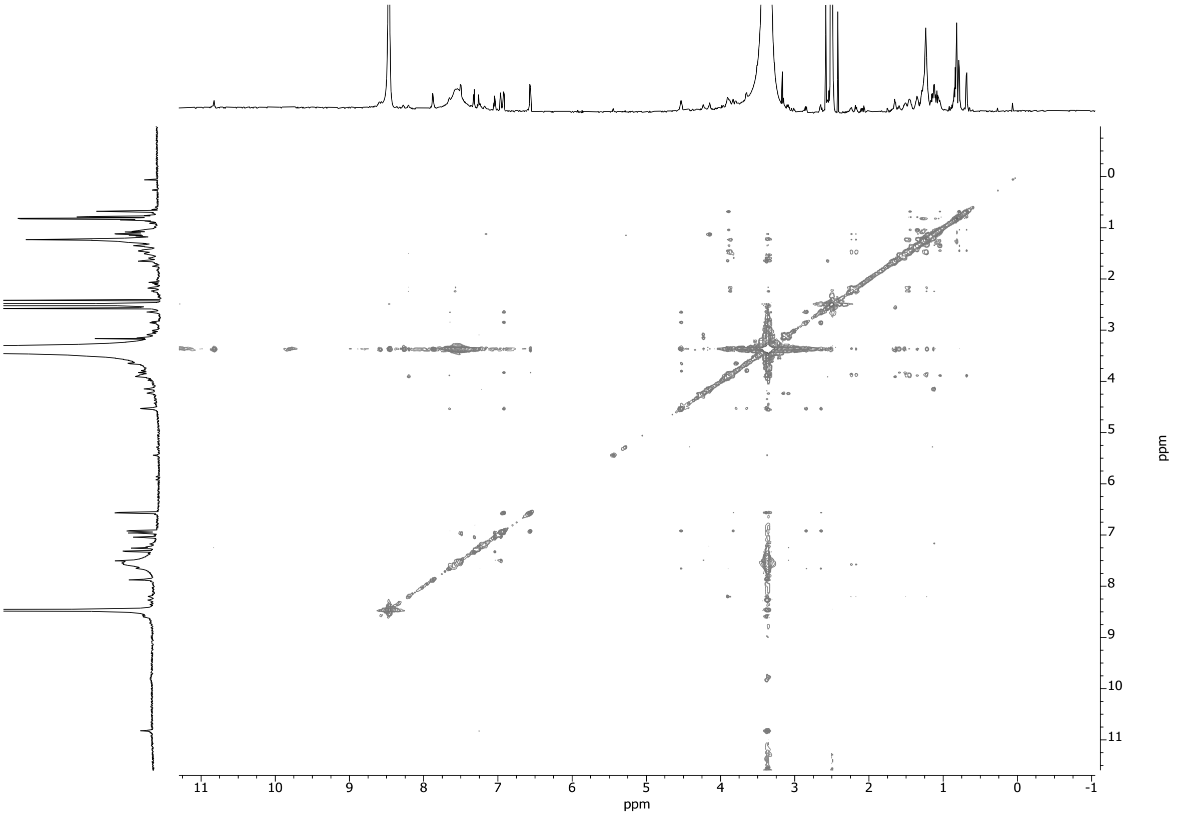

Figure S18. ¹H-¹H NOESY spectrum of **2** (850 MHz, in DMSO-*d₆*).

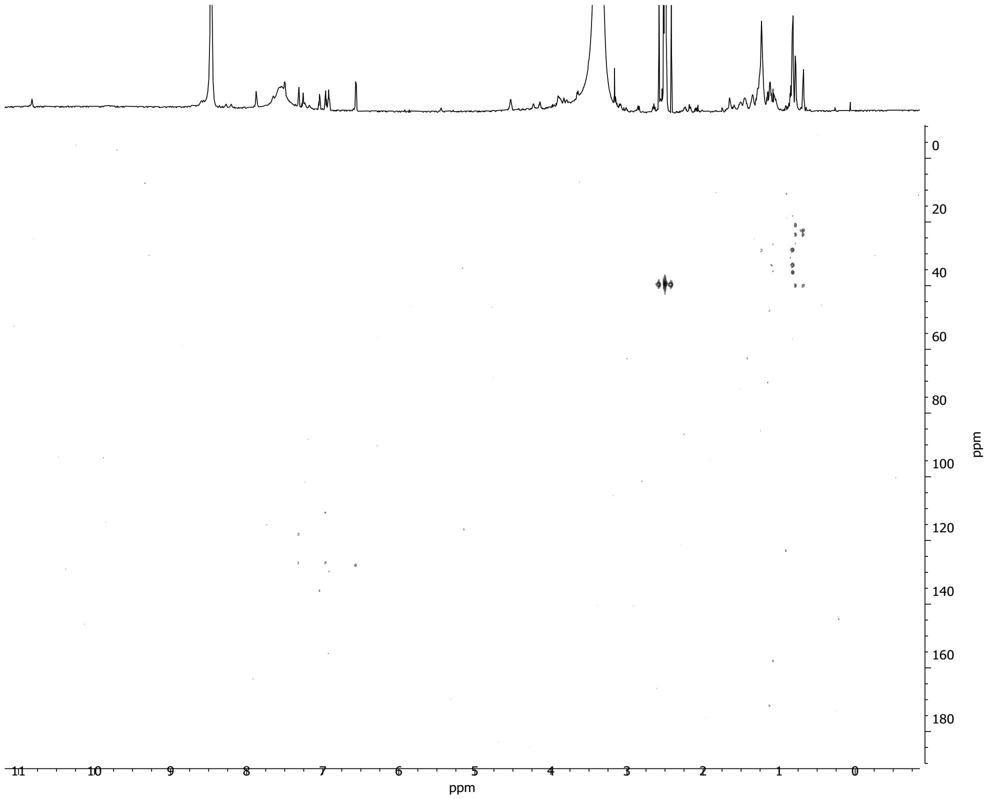

Figure S19. ^{1}H-^{13}C HMBC spectrum of **2** (850 MHz, in DMSO-d_6).

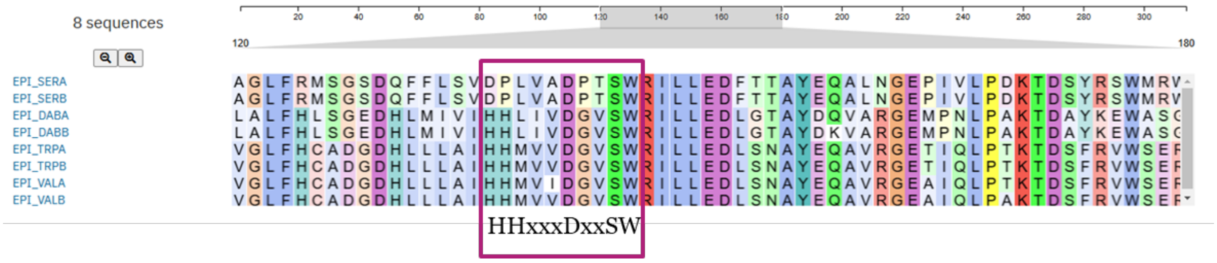

Figure S20. Clustal2 multiple sequence alignment of the epimerization domains of the paenilipoheptin A and B BGCs from *Paenibacillus* sp. JJ-21 and *Paenibacillus* sp. JJ-1722, respectively.

References

(1) O'Leary, N. A.; Wright, M. W.; Brister, J. R.; Ciufo, S.; Haddad, D.; McVeigh, R.; Rajput, B.; Robbertse, B.; Smith-White, B.; Ako-Adjei, D.; et al. Reference sequence (RefSeq) database at NCBI: current status, taxonomic expansion, and functional annotation. *Nucleic Acids Res.* **2016**, *44* (D1), D733-745. DOI: 10.1093/nar/gkv1189.

(2) Navarro-Muñoz, J. C.; Selem-Mojica, N.; Mullowney, M. W.; Kautsar, S. A.; Tryon, J. H.; Parkinson, E. I.; De Los Santos, E. L. C.; Yeong, M.; Cruz-Morales, P.; Abubucker, S.; et al. A computational framework to explore large-scale biosynthetic diversity. *Nat. Chem. Biol.* **2020**, *16* (1), 60-68. DOI: 10.1038/s41589-019-0400-9.

(3) Blin, K.; Shaw, S.; Kloosterman, A. M.; Charlop-Powers, Z.; van Wezel, G. P.; Medema, Marnix H.; Weber, T. antiSMASH 6.0: improving cluster detection and comparison capabilities. *Nucleic Acids Res* **2021**, *49* (W1), W29-W35. DOI: 10.1093/nar/gkab335.

(4) Shannon, P.; Markiel, A.; Ozier, O.; Baliga, N.; Wang, J.; Ramage, D.; Amin, N.; Schwikowski, B.; Ideker, T. Cytoscape: a software environment for integrated models of biomolecular interaction networks. *Genome Res.* **2003**, *13* (11), 2498-2504. DOI: 10.1101/gr.1239303.

(5) Lane, D. *16S/23S rRNA sequencing*; Wiley, 1991.

(6) Nguyen, D. D.; Melnik, A. V.; Koyama, N.; Lu, X.; Schorn, M.; Fang, J.; Aguinaldo, K.; Lincecum, T. L.; Ghequire, M. G. K.; Carrion, V. J.; et al. Indexing the *Pseudomonas* specialized metabolome enabled the discovery of poaeamide B and the bananamides. *Nat. Microbiol.* **2016**, *2* (1), 16197. DOI: 10.1038/nmicrobiol.2016.197.

(7) Vater, J.; Herfort, S.; Doellinger, J.; Weydmann, M.; Borriss, R.; Lasch, P. Genome mining of the lipopeptide biosynthesis of *Paenibacillus polymyxa* E681 in combination with mass spectrometry: discovery of the lipoheptapeptide paenilipoheptin. *ChemBioChem* **2018**, *19* (7), 744-753. DOI: 10.1002/cbic.201700615.

(8) Kieser, T., Bibb, M. J., Buttner, M. J., Chater, K. F. & Hopwood, D. A. *Practical Streptomyces genetics*; John Innes Foundation, 2000.

(9) Chin, C. A. O.; Peluso, P.; Sedlazeck, F. A.-O.; Nattestad, M.; Concepcion, G. T.; Clum, A.; Dunn, C. A.-O.; O'Malley, R.; Figueroa-Balderas, R.; Morales-Cruz, A.; et al. Phased diploid genome assembly with single-molecule real-time sequencing. *Nat. Methods* **2016**, *13*, 1050–1054. DOI: 10.1038/nmeth.4035.

(10) Blin, K.; Shaw, S.; Augustijn, H. E.; Reitz, Z. L.; Biermann, F.; Alanjary, M.; Fetter, A.; Terlouw, B. R.; Metcalf, W. W.; Helfrich, E. J. N.; et al. antiSMASH 7.0: new and improved predictions for detection, regulation, chemical structures and visualisation. *Nucleic Acids Res.* **2023**, *51* (W1), W46-W50. DOI: 10.1093/nar/gkad344.

(11) van Bergeijk, D. A.; Elsayed, S. S.; Du, C.; Santiago, I. N.; Roseboom, A. M.; Zhang, L.; Carrión, V. J.; Spaink, H. P.; van Wezel, G. P. The ubiquitous catechol moiety elicits siderophore and angucycline production in *Streptomyces*. *Commun. Chem.* **2022**, *5* (14). DOI: 10.1038/s42004-022-00632-4.

(12) Pluskal, T.; Castillo, S.; Villar-Briones, A.; Oresic, M. MZmine 2: modular framework for processing, visualizing, and analyzing mass spectrometry-based molecular profile data. *BMC Bioinformatics* **2010**, *11*, 395. DOI: 10.1186/1471-2105-11-395.

(13) Wang, M.; Carver, J. J.; Phelan, V. V.; Sanchez, L. M.; Garg, N.; Peng, Y.; Nguyen, D. D.; Watrous, J.; Kapono, C. A.; Luzzatto-Knaan, T.; et al. Sharing and community curation of mass spectrometry data with Global Natural Products Social Molecular Networking. *Nat. Biotechnol.* **2016**, *34* (8), 828-837. DOI: 10.1038/nbt.3597.

(14) Marfey, P. Determination of D-amino acids. II. Use of a bifunctional reagent, 1, 5-difluoro-2, 4-dinitrobenzene. *Carlsberg Res Commun* **1984**, *49*, 591-596. DOI: 10.1007/BF02908688.

(15) Lysenko, V.; Son, S.; Theriault, M. E.; Slingerland, C. J.; Hauk, G.; Cleenewerk, L.; Speer, A.; Berger, J. M.; Lewis, K.; Martin, N. I. Total synthesis and structural reassignment of the antitubercular natural product evybactin. *Chem. Eur. J.* **2024**, DOI: 10.1002/chem.202403767.
